# Anticipatory attentional avoidance in learned threat associations

**DOI:** 10.1007/s00426-026-02342-1

**Published:** 2026-07-15

**Authors:** Botond László Kiss, Giovanna C. Del Sordo, Michael C. Hout, Ferenc Kocsor, Andras N. Zsido

**Affiliations:** 1 Institute of Psychology, University of Pécs, Pécs, Hungary; 2 Szentágothai Research Centre, University of Pécs, Pécs, Hungary; 3 Psychology Department, New Mexico State University, Las Cruces, USA; 4 Kinesiology Department, New Mexico State University, Las Cruces, USA; 5 STEM+ Education Research Institute, New Mexico State University, Las Cruces, USA; 6 Research Centre for Contemporary Challenges, University of Pécs, Pécs, Hungary; 7 Institute of Psychology, University of Pécs, 6. Ifjusag Street, Pécs, Baranya, H 7624 Hungary

## Abstract

The processing of threatening content tends to take priority over neutral or other emotional stimuli. Compared to other attentional biases (in response to threat), less research has been dedicated to understanding anticipatory attentional avoidance, and existing studies on this topic have yielded mixed results. In this study, we investigated anticipatory attentional avoidance, focusing on how predictive cues associated with threatening stimuli influence attentional processing before the actual threat appears. We used a cued visual probe task, in which a neutral cue (onset times: 100, 500, or 1000ms) predicted the location of a threatening stimulus. Across two experiments, we examined both behavioral and eye-tracking measures to capture avoidance patterns. In Experiment 1 (behavioral: *N* = 33; eye-tracking: *N* = 23), predictive cues appeared in the same location as the subsequent target (i.e., in the corners of the screen), while in Experiment 2 (behavioral: *N* = 51; eye-tracking: *N* = 22), predictive cues were presented centrally, separating cue and target locations. Our results showed that participants associated the threatening content with the predictive cue and learned to avoid attending to the spatial position of the threat-related stimuli. This avoidance was reflected in slower reaction times and gaze patterns that shifted away from the threat-predictive cues during presentation, particularly at longer cue presentation times. However, we found no differences in the onset of gaze shifts to the target, suggesting that avoidance occurs primarily during early anticipatory stages. These findings suggest that anticipatory attentional avoidance may appear as a distinct process that could emerge in response to learned threat associations.

**Supplementary Information:**

The online version contains supplementary material available at https://doi.org/10.1007/s00426-026-02342-1.

## Introduction

Over the past few decades, several studies have shown that attention is biased towards threatening content, with threat-related information taking processing priority over neutral or other emotional stimuli (Cisler et al., 2009; Cisler & Koster, 2010; Csatho et al., 2008; March et al., 2017; McNally, 2019; Zsidó et al., 2017). According to the attentional bias toward threat (ABT) framework, the presence of a threat can result in three well-defined attentional biases: attentional grab, delayed disengagement, and avoidance (Cisler & Koster, 2010; Fox et al., 2001 a; Koster et al. 2006a, b). These attentional biases can be best described as resulting from two core attentional processes: stimulus-driven orientation (governed by the salience network, which includes the amygdala, insula, and frontal regions), and goal-directed attention (regulated by the executive control network, which spans frontal and parietal areas) (Dolcos et al., 2020; Mogg & Bradley, 2018). The salience network automatically directs attention toward visually salient objects (such as threats), potentially overwriting other ongoing tasks or behaviors. In contrast, the executive control network works to maintain task performance by inhibiting threat processing, in order to allow a fuller scanning of the visual field.

As these processes align with the definitions of attentional grab and delayed disengagement, a large number of studies have investigated and demonstrated these mechanisms in clinical, subclinical, and healthy populations (Bar-Haim et al., 2007; Keogh & French, 2001; Mathews & MacLeod, 2002; Mogg & Bradley, 1998). Previous research has consistently demonstrated the appearance of attentional biases toward different threat-related stimuli, including evolutionarily relevant threats, such as snakes and spiders (Luo et al., 2015; Miltner et al., 2004; Öhman et al., 2001), socially relevant threats, such as angry or fearful faces (Amir et al., 2003; Wieser et al., 2018; Zsidó et al., 2024), and modern threats such as guns and medical procedures (Armstrong et al., 2013; Behlau et al., 2024; Blanchette, 2006; Zsido et al., 2019). Specifically, attentional grab refers to the automatic capture of attention by a threatening stimulus, enhancing the ability to detect a potential threat (Öhman, 1986; Öhman et al., 2001; Stefanics et al., 2012). Once attention is drawn to the threat, delayed disengagement occurs, making it more difficult to redirect attention to another object (Carlson et al., 2009; Cisler & Olatunji, 2010; Koster et al., 2004). While these two attentional biases are well-documented phenomena, results related to the third bias, attentional avoidance, are inconsistent. Consequently, despite its potential importance in understanding phobic behavior, attentional avoidance has yet to be clearly defined. There are still open questions regarding its existence, characteristics, and whether it is distinct from other attentional biases.

Avoidance refers to the process of directing attention to a location away from the position of the threatening stimulus (Chen et al. 2002; Garner et al. 2006a; Koster et al., 2006b; McNally, 2019). Findings regarding this bias remain limited (Cisler & Koster, 2010), as its alignment to the other two core attentional processes is perhaps less intuitive. Previously, attentional avoidance has been mainly investigated using spatial cuing tasks, such as the dot-probe paradigm (Cisler & Koster, 2010; MacLeod et al., 1986; Mogg & Bradley, 1999). In these tasks, salient and neutral images are used as cues that precede the presentation of the neutral targets, and these cues are generally presented for short durations (100–500 ms). Past studies have yielded mixed results, likely because the paradigms used may not be fully suited to studying avoidance due to inherent design limitations.

The first design issue is that, according to the ABT framework and prior empirical evidence, longer cue presentation times (500–1000 ms) may be necessary for avoidance to occur (Chen et al. 2002; Garner et al. 2006b; Koster et al. 2005; Mogg et al. 2004a, b). However, on this point too, prior findings remain inconsistent: while some studies have demonstrated avoidance at shorter presentation times (Sagliano et al., 2014; Waters et al., 2007), others have found avoidance at presentation times above 1000ms (Bradley et al., 1998; Mogg et al., 1997). One possible explanation for this delayed onset is that avoidance may not be a distinct attentional bias but rather a secondary process that only occurs after another attentional bias has taken place (Koster et al., 2005).

The second issue relates to the use of spatial cueing tasks, where multiple attentional processes occur before the one identified as avoidance, which typically emerges at a later stage (i.e., after 500–1000 ms). These tasks compare congruent trials (where the target appears in the same location as the salient cue) and incongruent trials (where the target appears in the opposite location). However, they lack a true baseline condition against which avoidance behavior can be directly compared (Cisler et al., 2009). Therefore, the definition of attentional avoidance has been largely driven by these empirical observations rather than theoretical considerations. It is still unclear whether attentional avoidance is a primary attentional bias (such as attentional capture or grab), or rather a secondary attentional bias (that appears after attentional capture or grab). To resolve these contradictory results, we aimed to investigate a form of avoidance that occurs prior to the appearance of threatening content. By focusing on anticipatory avoidance, our approach reduces the chance of participants developing other attentional biases and establishes a clear baseline condition for studying avoidance as an independent process.

### Anticipatory attentional bias

Past studies have routinely measured attentional biases in the presence of a threat, but only a few have explored the bias that occurs when anticipating an encounter with a feared object. A lack of connection between clinical phobia research and threat-focused cognitive experiments may explain this gap. Anticipatory attentional bias describes the phenomenon by which an attentional bias manifests when the salient stimulus is not yet present but is anticipated to occur (Bastiaansen & Brunia, 2001). Specific phobias exhibit this anticipatory effect as a form of avoidance behavior (Brinkmann et al., 2017). Anxiety not only occurs when individuals interact with the object they fear, but also when they think about it or anticipate a situation where they might encounter it. For instance, spider-fearful individuals may avoid a park, which is a natural habitat for arthropods, while those fearful of blood-injection-injury-related stimuli may delay or completely avoid making an appointment for medical screening or blood draws (Birkás et al., 2023; Kiss et al., 2022). Furthermore, previous behavioral studies have shown that attentional avoidance occurs when medical stimuli and disgust-related images are presented (Armstrong et al., 2013; Buodo et al., 2010). Attentional avoidance may also play a crucial role in facilitating exploration of the environment and acquiring relevant information about a current situation (Koster et al., 2006b; Pflugshaupt et al., 2005). An early anticipatory form of this avoidance behavior can be achieved by using a modified cueing paradigm, such as the cued Visual Probe Task (cVPT) (Gladwin, 2017; Gladwin & Vink, 2018).

The cVPT is a modified dot-probe task providing a solution to investigating avoidance behavior related to a certain type of stimulus (Basanovic, 2024; Gladwin, 2017). One key modification compared to a standard dot-probe task is that neutral cues are used to indicate the possible location of emotional stimuli, thereby providing an opportunity to associate (learn) the cue in relation to the appearance of a certain type of stimuli. These same neutral cues then precede the onset of both the target and the foil, effectively separating the probe detection task from the presentation of the emotionally-charged stimulus. As a result, the task consists of two different trial types: picture trials and stimulus trials. In the picture trials, the emotionally salient and neutral pictures are presented, and each is associated with a certain neutral cue. In the stimulus trial, the target and distractor stimuli are presented after the neutral cues. The target stimulus can appear at the location of the threat-related cue (congruent trial) or at the location opposite to it (incongruent trial). The degree of anticipatory attentional bias towards threat is measured by comparing reaction times in congruent and incongruent trials; avoidance behavior is indicated by faster responses to targets appearing in the opposite location of the threat-related neutral cue. Previous studies using the cVPT paradigm have primarily used socially threatening stimuli (e.g. emotional faces), and these studies have typically reported attentional capture toward the cues. This pattern may reflect the specific characteristics of the threatening stimuli (e.g., accurate identification of the emotion appearing on the face). However, most recent work has demonstrated anxiety-related avoidance of negative cues (Basanovic, 2024), suggesting that this paradigm can trigger avoidance behaviour.

### Present study

**Fig. 1:**
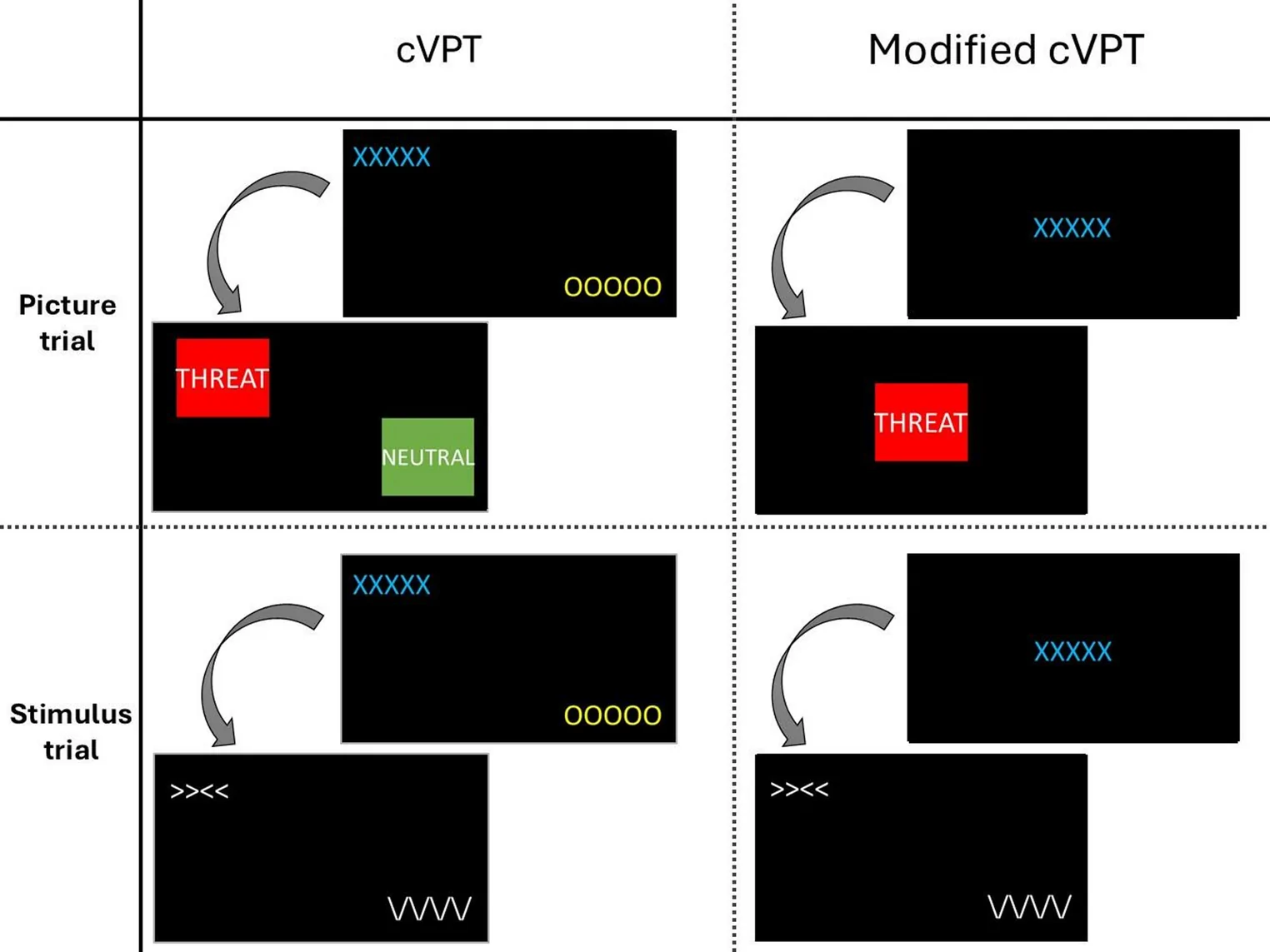
Schematic presentation of the original cued Visual Probe Task (cVPT, left columns), and our modified version (right column). In the Picture trial of the cVPT (top-left), participants first view two letter strings (“XXXXX” and “OOOOO”), followed by the presentation of two pictures (threatening and neutral). In the Modified cVPT (top-right), after presenting a single letter string (“XXXXX” or “OOOOO”), only one image (threatening or neutral) is displayed. In the Stimulus trial of the cVPT (bottom-left), the letter string is followed by a target stimulus (>><<) and a distractor stimulus (“VVVVV”), while in the Modified cVPT (bottom-right), only one letter string is presented, but the target and the distractor stimuli are shown in the same manner as in the cVPT arrangement

We propose a modification to this paradigm (see Fig. 1) in which picture trials and neutral predictive cues are presented at a location (e.g., the center of the screen) that is spatially distinct from the target and distractor locations (e.g., which remain in the corners of the screen). This design departs from the congruent-incongruent structure typical of dot-probe-like tasks and allows for the testing of the anticipatory avoidance effect independently of these conditions. If the appearance of the threat-related neutral cue indeed triggers avoidance behavior, participants should locate the subsequent target more quickly when the cue signals a threat than when it does not. This effect would arise because participants anticipate the threat’s appearance in the center of the screen and then actively try to avoid that spatial position. By implementing these changes to the cVPT paradigm, (1) no emotional pictures may induce a priori attentional biases, while participants may still engage in an anticipatory attentional process based on threat expectation; and (2) this allows the attentional bias to be driven by stimulus *category* rather than the specific stimulus *content* (Gladwin & Vink, 2020), thereby enhancing the reliability of the results (Gladwin, 2019).

Measuring eye movements is a common way to complement behavioral measures (e.g. reaction time) by tracking attentional processes over time and providing additional information about the development of attentional biases. Eye-tracking studies consistently show that threatening or negative stimuli capture attention more rapidly and are more difficult to disengage from than neutral stimuli, as demonstrated in visual search and dot-probe paradigms (Fox et al., 2001; Waechter et al., 2014). However, eye-tracking data is often analyzed by averaging measures across the entire trial, rather than examining how attentional shifts unfold over time. Because eye movements occur rapidly, previous studies have suggested that, in some cases, covert rather than overt attention influences processing (Calvo et al., 2015; Cave & Batty, 2006). Thus, an individual not directly attending to emotionally charged (threatening) content may still show some degree of attentional bias. Trial-level averaging may therefore overlook fast, transient eye movements. Therefore, analyses that examine gaze dynamics within individual trials are better suited to capturing these processes (Godwin et al., 2021). Accordingly, we tracked gaze distance from the anticipated threat location over time and estimated the onset of attentional shifts toward the target cue in both congruent and incongruent conditions.

In the present study, we investigated whether anticipatory avoidance of threatening content can be captured in a visual probe task using both behavioral and eye-tracking measures. Across two experiments, we used a modified version of the cVPT paradigm and manipulated the valence (threatening or neutral) of the associated images. In Experiment 1, predictor cues and their corresponding content (threat and neutral) appeared in the same spatial location as the target, in the corner of the screen. In Experiment 2, to further explore the attentional avoidance bias, neutral predictor cues and their corresponding threatening and neutral content appeared in the center of the screen, while the target appeared in one of the corners.

## Experiment 1

In Experiment 1a, participants performed the modified cued Visual Probe Task (cVPT), in which they were required to locate a target cue while ignoring a similar distractor appearing on the opposite side of the screen. The target-distractor pair was always preceded by predictive cues (letter strings) associated with either neutral or threatening content. We manipulated the presentation time of these predictor cues (100ms, 500ms or 1000ms). The target stimulus appeared either in the position of the predictor cue associated with threatening content (which henceforth will be referred to as *congruent*) or in the position of the predictor cue associated with neutral content (which will be referred to as *incongruent*). Experiment 1b was a replication and extension of Experiment 1a, incorporating eye-movements to monitor participants’ gaze behavior.

We hypothesized that participants would tend to avoid the location of a potential threat, as signaled by the associated neutral cue. Our hypotheses were the following: (H1a) participants would be slower to locate the target in the congruent condition (when the threat-related cue appeared in the target’s location) compared to the incongruent condition (when the threat-related cue appeared in the opposite location); (H2) over time, participants’ gaze would be located further away from the location of the possible threat; and (H3a) the offset time of attentional shifts toward the target would be delayed for congruent compared to incongruent trials.

## Methods

### Participants

The required sample size for Experiment 1a was determined using G*Power software (version 3.1.9.7; Faul et al., 2007). For the within‑subjects Congruency (2) × Presentation Time (3) interaction in a 2 × 3 repeated‑measures ANOVA (rmANOVA), we used conservative parameters for the estimation (f = 0.25, 1-β = 0.95, *r* =.5, α = 0.05), which indicated a minimum required sample size of 28. Data collection was carried out in one-week increments until the required sample size was reached or exceeded. The final sample consisted of 33 undergraduate students (22 females, mean age = 22.3, SD = 3.69). For Experiment 1b, the required sample size for the rmANOVA was determined based on the effect size observed in Experiment 1a (f = 0.49, 1-β = 0.95, *r* =.5, α = 0.05). While the analysis suggested a minimum sample size of 9 participants, we aimed for a larger sample due to the inclusion of the eye-tracking measures, to account for potential data loss due to technical issues (e.g., poor calibration, excessive motion artifacts). Data collection was carried out in one-week increments until the required sample size was reached or exceeded. The final sample consisted of 23 undergraduate students (16 females, mean age = 20.5, SD = 1.31). For the analysis of eye-tracking data, we ultimately did not rely on rmANOVA, but instead used linear mixed-effects (LME) models, which allowed us to treat time as a continuous variable in the models. Since no a priori sample size estimation were performed for this analysis, we performed a post hoc power calculation using the “simr” package (Green & MacLeod, 2016). The results suggested that the sample size was adequate for the gaze-distance outcome (power = 78.0%; 95% CI: [75.30, 80.53]). Given ongoing concerns about the interpretability of post hoc power analyses, we also report 95% confidence intervals for all LME estimates (Heckman et al., 2022). Importantly, neither the a priori nor the post-hoc power analyses account for the number of repeated observations. In the present study, each participant contributed a large number of trials, which increases the precision and reliability of the estimates.

All participants reported normal or corrected-to-normal vision. Based on self-report, none of the participants in our study had been diagnosed with or treated for phobia or other psychiatric disorders. All participants were recruited through university mailing lists and were paid 5000 HUF for their participation. Our research was approved by the Hungarian United Ethical Review Committee for Research in Psychology (approval number: EPKEB 2024-084) and was conducted following the guidelines of the World Medical Association Code of Ethics (Declaration of Helsinki). All participants were informed, and written informed consent was obtained.

### Experimental stimuli

The images used in the study were selected from various image databases^1^. All selected images had been previously rated by an independent sample in a previous study along four dimensions: valence, arousal, threat, and disgust (Kiss et al., 2022). For detailed descriptive data of these ratings, see Table 1. A total of 72 images were selected, comprising 36 neutral images (e.g., objects, neutral faces, hands) and 36 threatening images (e.g., syringe, surgery, blood draw, injury). All images were 350 × 262 pixels. The same set of stimuli was used for both Experiments 1a and 1b.

**Table 1:** Means and standard deviations (SD) for the rating dimensions (valence, arousal, threat, disgust) of the threatening and the neutral pictures used in the study

	Valence		Arousal		Threat		Disgust	
	Mean	SD	Mean	SD	Mean	SD	Mean	SD
Threat	3.00	0.48	4.82	0.80	4.18	0.77	3.74	1.23
Neutral	5.48	0.49	2.16	0.2	1.58	0.19	1.40	0.09

The pictures were rated using 9-point Likert-type scales. Higher scores indicated greater unpleasantness, arousal, threat, and disgust

### Procedure and apparatus

In Experiment 1a, the task was performed in a quiet room, accommodating up to 10 participants at a time, each seated separately. The stimuli were presented on a 24-inch TFT color monitor, with a display resolution of 1440 × 990 pixels, a 16:9 aspect ratio, a refresh rate of 60 Hz, and a color depth of 16.7 M colors. The experiment was implemented using PsychoPy software version 2022.2.4 (Peirce et al., 2019) to present the trials and collect participants’ responses. Upon arrival at the laboratory, participants were seated approximately 60 cm from the monitor and were provided with both oral and written instructions about the task. The experiment followed a 2 (Congruency: congruent, incongruent) x 3 (Predictor cue presentation time: 100, 500, 1000 ms) factorial design.

**Fig. 2:**
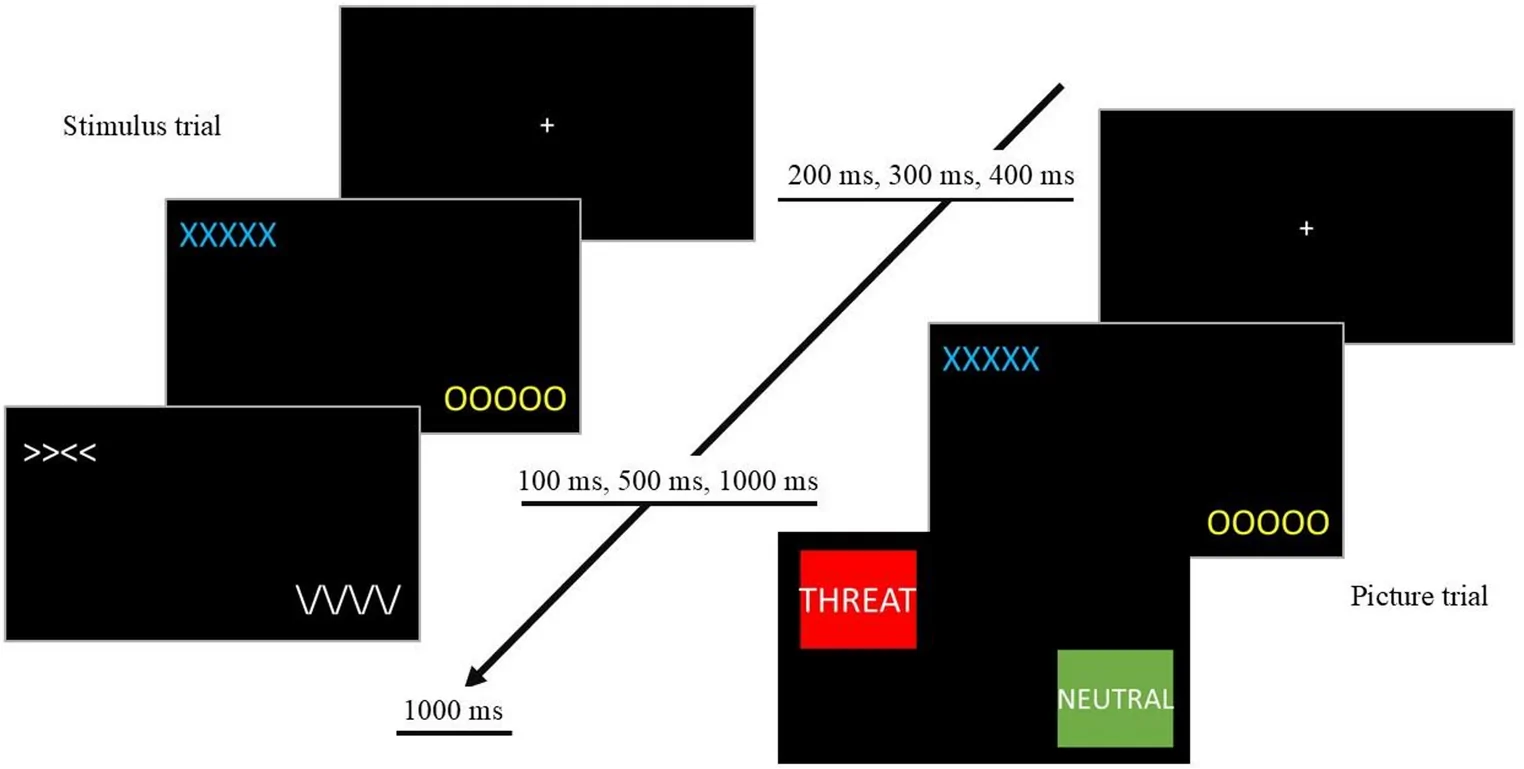
Trial procedures for the cVPT task in Experiment 1. In stimulus trials, the target (>><<) and foil (/\/\/\ or \/\/\/) stimuli were presented after the predictive stimuli. In picture trials, after presenting a pair of predictive stimuli diagonally across the screen, a pair of images (threat and neutral) were presented in the position associated with the predictive stimuli

The experimental paradigm consisted of two trial types, picture and stimulus trials (see Fig. 2 for a detailed overview), which were randomly alternated throughout the session. For both trial types, each trial began with a fixation cross displayed for 200–400 ms, followed by two predictor cues presented diagonally on the screen for either 100 ms, 500 ms or 1000 ms. Predictor cues consisted of the letter string *OOOOO* in yellow (RGB values: [250, 250, 10]) or the letter string *XXXXX* in light blue (RGB values: [10, 250, 250]). The participants were informed about the task (i.e., which button to press in which situation), but were not informed about the association between the predictive cue and the images.

In *picture trials*, after the presentation of the predictor cues, a neutral or a threatening picture was shown in the same diagonal arrangement as the predictors for 1000 ms. For each participant, one type of letter string (e.g. ‘XXXXX’ or ‘OOOOO’) was consistently associated with threatening images, while the other was associated with neutral pictures. The specific predictor-picture association was randomized across participants. Participants were instructed to simply observe during these displays and not to press any keys. In *stimulus trials*, the target stimulus (>><<) and one of the foil stimuli (/\/\/\ or \/\/\/) were presented after the predictive stimulus. Participants’ task was to indicate the location of the target stimulus by pressing a key as quickly and accurately as possible. The keys assigned to each position were: R for the top left corner of the screen, F for the bottom left, I for the top right, and J for the bottom right. During the experiment, the trial types were randomly alternated. The fixation cross and predictive stimuli presentation time, and the location of the predictive cues were randomized across trials. The experiment consisted of 576 trials (evenly split across the two trial types), presented in a single testing session lasting approximately 25–30 min.

In Experiment 1b, the same procedure as in Experiment 1a was followed. The total number of trials was 288, and data were collected individually rather than in a group setting. We reduced the number of trials because the additional tasks involved in eye-tracking measurement (e.g., instrument setup, calibration) would have significantly increased the duration of the experiment, which would potentially cause participant fatigue. The experiment was presented on a 23-inch TFT color monitor with a resolution of 1920 × 1080 pixels, a 16:9 aspect ratio, a refresh rate of 60 Hz, and a color depth of 16.7 M colors. The experiment was presented using PsychoPy software (version 2022.2.4). Eye movements were recorded using a Tobii Pro TX300 eye-tracker at a sampling rate of 300 Hz. A nine-point calibration was performed prior to the experiment, and the calibration accuracy was assessed manually and repeated if the test fixation was found to be outside a radius of 2.25° around any of the five points. Behavioral responses were recorded using a Cedrus StimTracker. The session lasted approximately 30–45 min.

### Statistical analysis

In both Experiments 1a and 1b, median reaction times (RTs) were calculated for each participant across the different conditions. Median values were used instead of means as the median is less sensitive to sample variability (Bonamente, 2017). In Experiment 1b, eye-tracking measures were also analyzed, including: (1) gaze distance from the position of the threat-related predictor cue^2^, and (2) the latency between the target onset and the initiation of an eye movement toward the target location. Gaze distance was measured as the Euclidean distance (in pixels) between the threat-related predictor cue and the gaze position of the left eye, for each frame during the 150ms preceding the predictor cue offset. Movement onset time was estimated by identifying inflection points in the gaze trajectory. To do so, the data were first smoothed using an Exponentially Weighted Moving Average (EWMA) method with a 200ms time window. EWMA smoothing was designed to reduce short-term fluctuations in the data while preserving overall trends (Schat et al., 2024; Smit et al., 2023). A 200 ms sliding window was then applied to the smoothed data to identify inflection points. Inflection points were defined as the time points at which the increase or decrease in distance began to remain constant and did not return to previous levels in the following period. Smoothing was only used to determine inflection points. Prior to that, no smoothing was applied for distance comparisons. While all stimulus trials were included in the eye-tracking analysis, picture trials were only analyzed in relation to the predictor cues. In Experiments 1a and 1b, for the behavioral results, outlier trials were identified and removed, and defined as those exceeding the ± 2.5 standard deviations from the group median. This resulted in the removal of less than 5% of the data collected. Statistical analyses were conducted using Jamovi (version 2.3.18; Jamovi Team, 2021) for behavioral data and using RStudio (R Core Team, 2020) for eye-tracking data.

For the behavioral data in both Experiments 1a and 1b, we performed a 2 × 3 rm ANOVA with Congruency (Congruent, Incongruent) and Predictor cue presentation time (100, 500, 1000 ms) as within-subject factors. We used rmANOVAs per factor to follow up on the significant interaction between the two factors. In addition to *p*-values, we also estimated effect size using partial eta squared (η_p_^2^). Significant effects were followed by Tukey-corrected post hoc tests. The normality assumption was not violated, with absolute values of skewness and kurtosis below two for all factors. Only RTs from correct trials were analyzed, resulting in the exclusion of less than 5% of the collected data. Split-half reliability was assessed using Spearman-Brown correction, which indicated good reliability (see Supplementary Material 2.)

For eye-tracking data (Experiment 1b), two LMEs were computed. In the first model, gaze distance from the threat-related predictor cue served as the dependent variable, with Presentation Time of the predictor cue (100, 500, 1000ms), and Time (continuous; from 150ms prior to the predictor cue offset) as fixed effects. In the second model, the time of the first eye movement onset was used as the dependent variable, and Predictor Cue (Congruent, Incongruent) and Presentation Time (100, 500, 1000ms) were fixed effects. All models included Participants as a random effect to account for individual variability. LMEs were computed using the “lme4” package (Bates et al., 2015) and the “emmeans” package (Lenth et al., 2025) for post hoc tests. Models were fit using maximum likelihood estimation, and significance testing for fixed effects was performed using Satterthwaite’s approximation for degrees of freedom. This approach appropriately handles repeated measures data by considering both within-subject and between-subject variability. The inclusion of random effects was justified by comparing models with and without the random intercept for participants using likelihood ratio tests (Luke, 2017). Our datasets (including computed study variables) are available on the Open Science Framework: https://osf.io/ej2g7/.

## Results

### Behavioral results

**Fig. 3:**
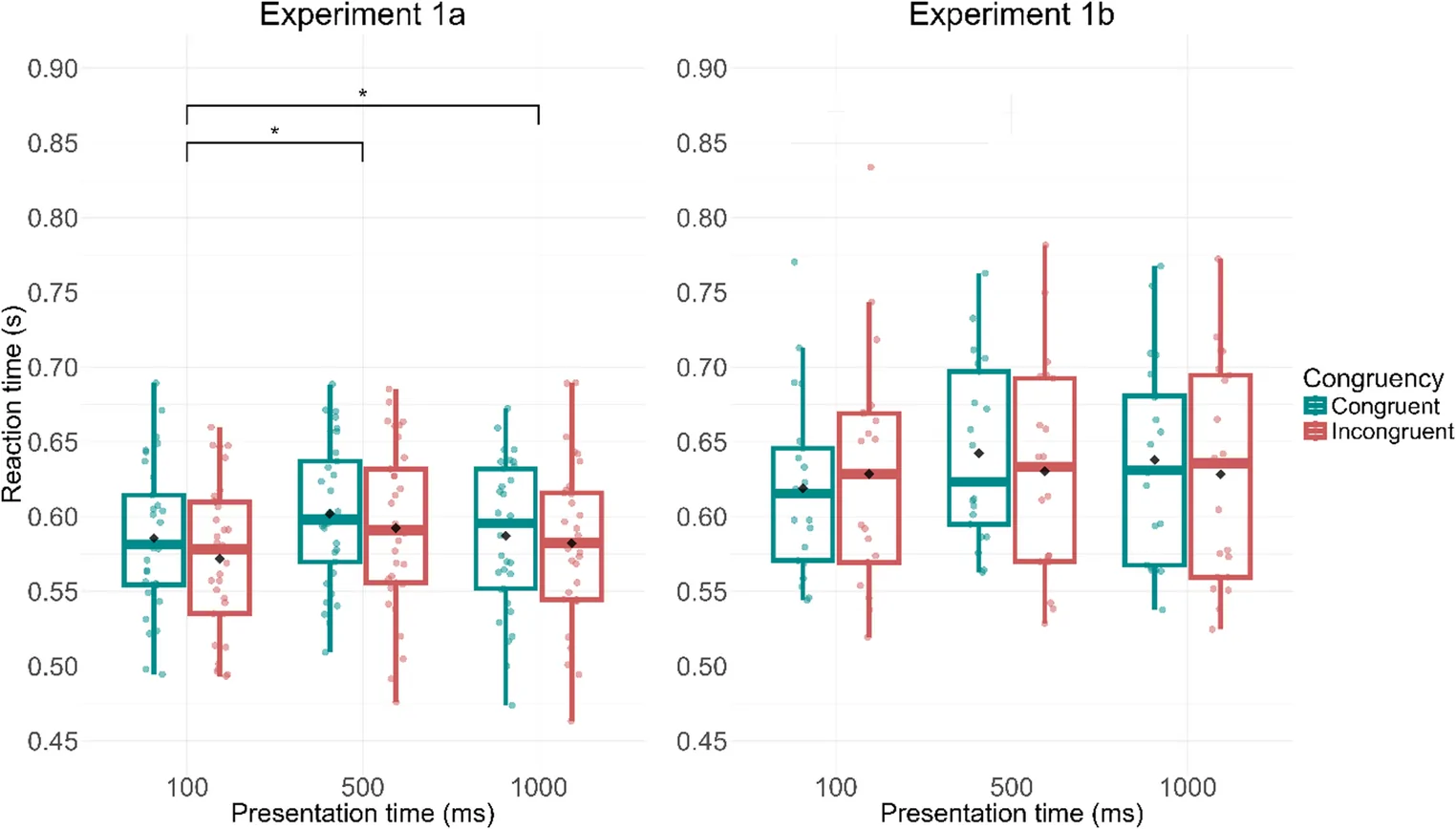
Reaction times (in seconds) for Experiment 1a (left panel) and for Experiment 1b (right panel). Median reaction times (Y-axis) are presented as a function of predictor cue presentation time (X-axis) and Congruency. The horizontal middle lines within the boxes indicate the median value, while the black diamonds represent the mean value. The ends of the box indicate the upper (Q3) and lower (Q1) quartiles. The lines extending from the boxes show the range of values within Q3 + 1.5 × IQR to Q1–1.5 × IQR, representing the highest and lowest values, excluding outliers. Transparent dots show the median reaction time for each individual. *p<.001

Reaction times in Experiment 1a were analyzed to test the hypothesis (H1a) predicting that participants would locate the target more slowly in congruent trials compared to incongruent trials. Descriptive statistics for these comparisons are presented in Fig. 3; and detailed statistical results are presented in Table 2. A significant main effect of Congruency was found, indicating that participants responded more slowly on congruent trials than on incongruent trials. There was also a significant main effect of Presentation Time, whereby RTs were significantly longer for the 500 ms condition compared to both short (100 ms) and long (1000 ms) presentation times. However, the interaction between Congruency and Presentation time was not significant. In line with our prediction, participants were slower in the congruent condition, possibly due to a tendency to avoid the location of the predictor cue that was signaling threatening content.

**Table 2:** Repeated measures ANOVAs and post hoc results for Experiment 1a and 1b for reaction times. Post hoc pairwise comparisons are reported only when the main effect or the interaction was significant. Significant main effects were followed up with Tukey-corrected pairwise comparisons, whereas significant interactions were examined using follow‑up ANOVAs

Effect	Comparison	df	F/t	*p*	η²*p*
*Experiment 1a*					
Congruency		1, 32	9.30	**0.005**	0.225
Presentation time		2, 64	13.78	**< 0.001**	0.301
	100–500	32	−4.135	**< 0.001**	
	100–1000	32	−0.896	0.647	
	500–1000	32	5.432	**< 0.001**	
Congruency ✻ Presentation time		2, 64	1.23	0.300	0.037
*Experiment 1b*					
Congruency		1,20	0.525	0.477	0.026
Presentation time		2,40	4.048	**0.025**	0.168
	100–500	20	−2.275	0.083	
	100–1000	20	−2.009	0.136	
	500–1000	20	0.975	0.601	
Congruency ✻ Presentation time		2,40	3.295	**0.047**	0.141
	Incongruent	2,40	0.094	0.910	0.005
	Congruent	2,40	5.51	**0.008**	0.216
	100–500	20	−2.561	**0.047**	
	100–1000	20	−3.222	**0.011**	
	500–1000	20	0.627	0.807	

Bolded p-values represent significant effects

RTs in Experiment 1b were analyzed to test the same hypothesis (H1a) concerning avoidance behavior (see Fig. 3; Table 2). In this experiment, the main effect of Congruency was not significant. Although there was a significant main effect of Presentation time, post hoc comparisons did not reveal any significant pairwise differences. However, a significant interaction between Congruency and Presentation time was observed. In the incongruent condition, RTs did not significantly differ across Presentation times. In contrast, in the congruent condition, participants responded more slowly when the predictor cues were presented for 100 ms compared to when they were presented for 500 or 1000 ms. No significant difference was found between the 500 ms and 1000 ms presentation times. Contrary to our prediction, participants were not generally slower in congruent compared to incongruent trials.

### Eye-tracking results

In Experiment 1b, gaze distance over time (from the location of the threat-related predictor cue) was analyzed to test the second hypothesis (H2), which predicted that participants’ gaze would be located further away from the location of the potential threat. Descriptive statistics for these comparisons are shown in Fig. 4, while Table 3 presents the statistical results. The fixed effects results indicated that the effect of the continuous variable Time Window was not significant, with a small estimate value. This indicated that, across all presentation times, Distance did not change systematically as the time window increased. In practical terms, Distance remained relatively stable across different time windows. The estimated gaze distance at time zero in the reference condition (100 ms presentation time) was 1103.07 pixels. Although the main LME model revealed a significant overall effect of Presentation Time, post hoc comparisons indicated no reliable differences between specific durations. Relative to the 100 ms condition, gaze distance was on average 26.1 pixels greater in the 500 ms condition and 25.5 pixels greater in the 1000 ms condition (*t*(14) = − 1.90, *p* =.174, and *t*(14) = − 1.67, *p* =.274, respectively). The comparison between the 500 and 1000 ms conditions likewise showed no difference, with only a 0.6 pixel change in gaze distance (*t*(10) = 0.03, *p* =.999). The interaction term showed a significant negative estimate, meaning that for 500ms, the slope of the relationship between Time Window and Distance was negative and significantly different from the reference condition (100ms). This suggested that for 500ms presentations, as the time window increased, the Distance decreased slightly, by about 0.167 pixels per unit increase in the time window. The interaction for 1000ms presentations was not significant, indicating that the slope of the relationship between Time Window and Distance was similar to the reference condition (100ms). Post hoc comparisons showed that there was a significant difference in the slope between the 500ms and 1000ms (*β* = − 0.178, *SE* = 0.066, *t*(4975) = −2.713, *p* =.018), suggesting that for 500ms presentations, as the time window increased, the Distance decreased slightly, by about 0.178 pixels per unit increase in the time window. Random effects indicated modest variability in baseline gaze distance but substantial individual differences in the effect of presentation time. Participants responded heterogeneously to longer display durations, with some showing greater gaze dispersion and others maintaining a consistent focus. These results highlight meaningful variability in individual viewing strategies beyond the average group trend.Fig. 4*Top panel*: Gaze distance from the threat-predictive cue over time as a function of cue presentation time (100, 500, 1000 ms). Each trace shows the mean distance (pixels) from cue onset (0 ms) to cue offset for the specified duration; larger values indicate gaze being located farther from the threat position. The 100 ms condition is shown in red, the 500 ms condition in green, and the 1000 ms condition in blue. *Bottom panel*: Visualization of the gaze distance (in pixels) from the location of the threat-related predictor cue across time, shown separately for the Presentation Time (in milliseconds) of the predictor cue (100 ms, 500 ms, 1000 ms).For this panel the time window corresponds to the 150 ms interval preceding cue offset. * *p*<.05; **p<.01; ***p<.001

**Figure:**
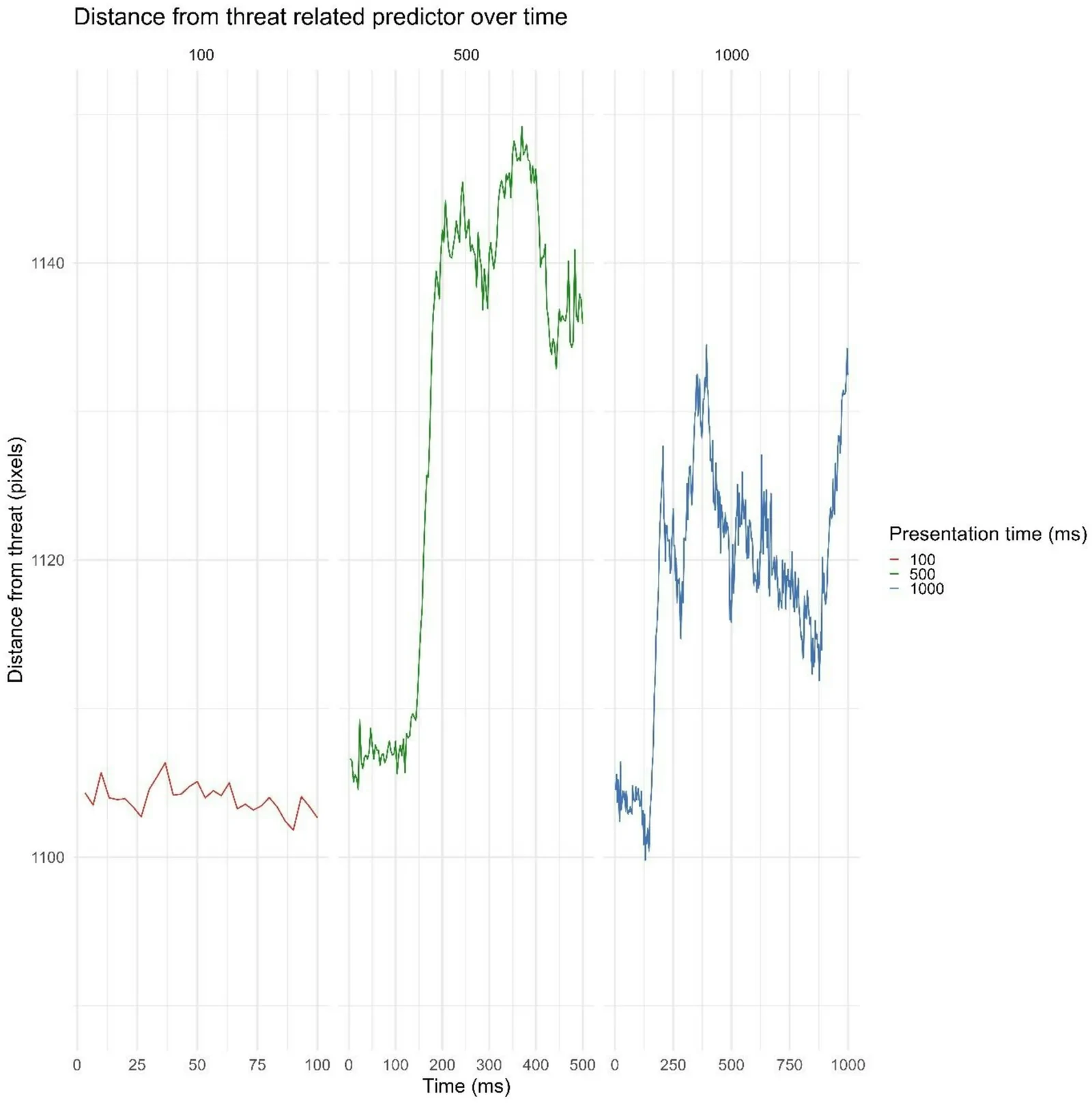

**Figure:**
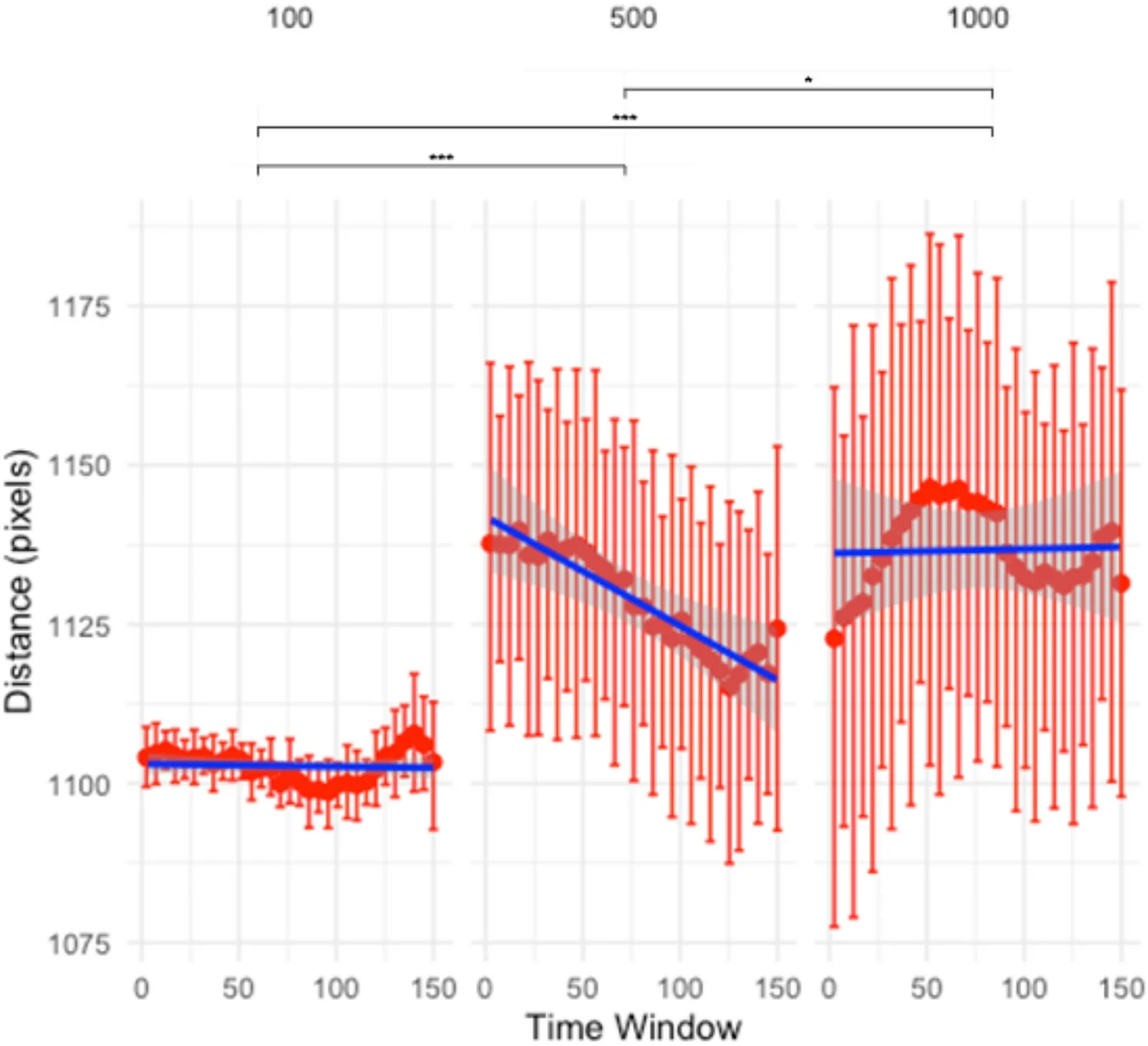

**Table 3:** Fixed effects of the linear mixed-effects model for gaze distance in experiment 1b

Fixed Effect	Estimate	CI 95%		df	t	*p*
		Lower	Upper			
Intercept	1103.08	1096.73	1109.42	103.87	339.26	< 0.001
Time window	−0.004	−0.07	0.06	4955	−0.12	0.903
Presentation Time (500 ms)	38.82	10.34	67.30	16.07	2.74	**0.014**
Presentation Time (1000 ms)	24.66	−10.80	57.50	9.29	1.55	0.154
Time window ✻ Presentation Time (500 ms)	−0.17	−0.26	−0.07	4955	−3.15	**0.001**
Time window ✻ Presentation Time (1000 ms)	0.01	−0.11	0.13	4955	0.18	0.858
Random Effect	**Random slopes**	**Variance**	**SD**	**Correlation**		
Participant	Intercept	25.85	5.08			
	Presentation time (500ms)	2746.44	52.41	−0.09		
	Presentation time (1000ms)	1905.93	43.66	−0.66	−0.16	
Residual		4336.38	65.85			

The intercept represents the reference condition, which includes the neutral-related predictor and the 100 ms presentation time. All other estimates should be interpreted relative to this baseline. Bolded p-values represent significant effects. Abbreviations: *df* degrees of freedom, *SE* standard error

Consistent with our hypothesis, participants’ gaze tended to remain further from the threat-related predictor cue than from the neutral-related predictor cue, particularly at longer presentation times.

**Fig. 5:**
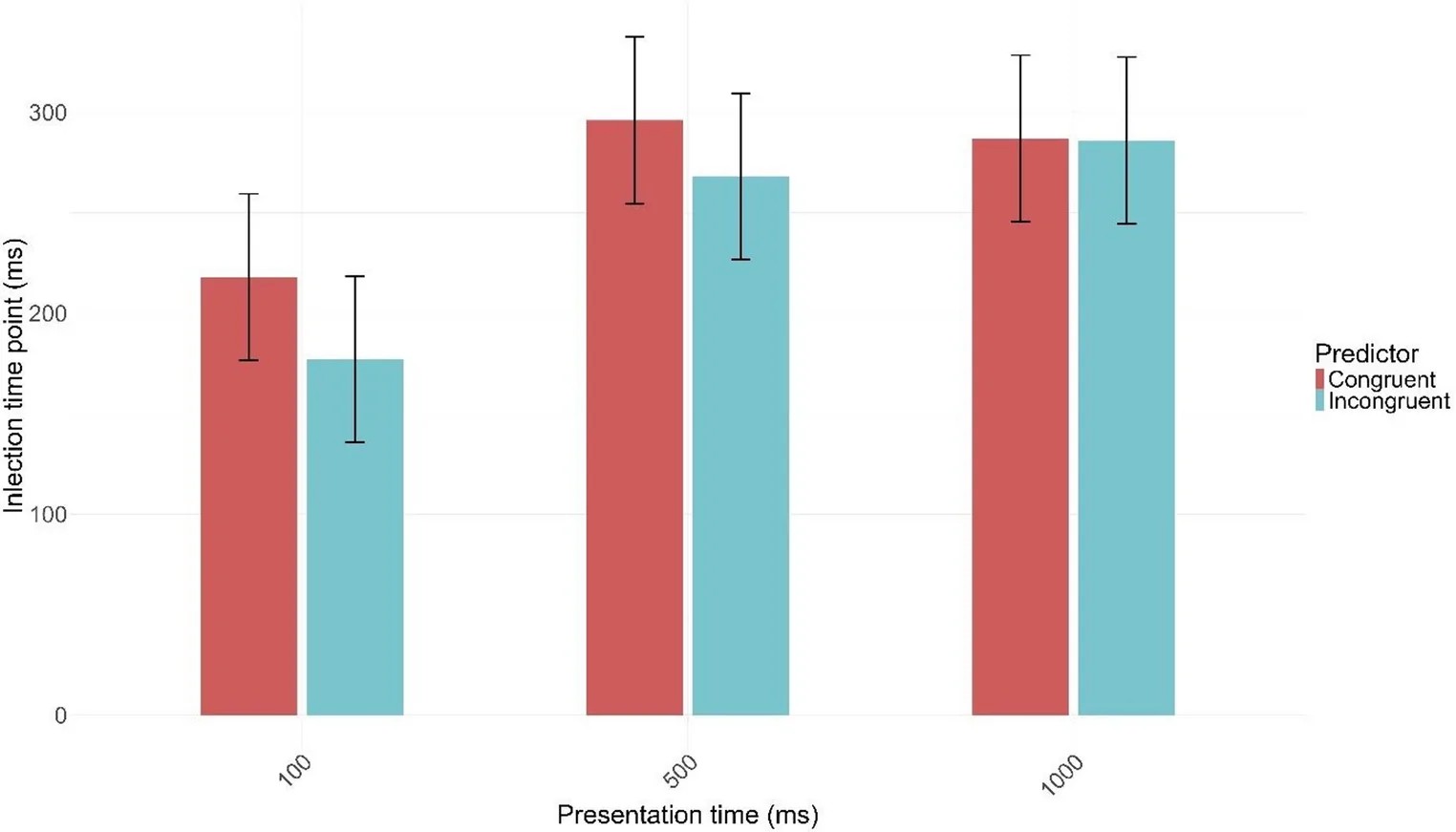
Inflection time point (in milliseconds) of the gaze movement toward the target stimuli as a function of presentation time and predictor congruency

Finally, in Experiment 1b, the timing of gaze movement onset was analyzed to test the third hypothesis (H3a), which predicted that attentional shifts toward the target would be delayed for congruent compared to incongruent trials. Descriptive statistics for these comparisons are presented in Fig. 5, and detailed statistical results are reported in Table 5. Fixed effects results indicated that the average inflection point (i.e., gaze initiation time) in the reference condition (congruent trials and 100 ms presentation time) was 217.66 ms. The Congruency (congruent vs. incongruent trials) factor showed a non-significant negative effect, suggesting that gaze initiation time was slightly slower (though not significantly) in the incongruent compared to the congruent condition. The presentation time for 500 ms and 1000 ms conditions both showed a positive trend, indicating a small increase in gaze initiation time compared to the 100 ms presentation time, though it was not significant. Interaction effects were tested between Congruency, and Presentation time. The interaction effect for the 500 ms condition was small and non-significant, as was the interaction for 1000 ms presentation time, indicating no significant interaction effects. Overall, the random effects indicate that participants differed modestly in their average performance but substantially in their responses to task and timing manipulations. Variability was largest for the 1000 ms presentation time (SD = 147.31), followed by the incongruent task type (SD = 87.02) and the 500 ms presentation time (SD = 71.23). The strong positive correlation between the 500 ms and 1000 ms slopes (*r* =.99) suggests that participants who showed larger effects at one duration tended to show similarly large effects at the other. The negative correlation between the intercept and Task Type slope (*r* = −.54) indicates that participants with higher baseline values tended to show smaller task-type differences.

Contrary to our prediction (H3a), these results suggest that neither Congruency (congruent vs. incongruent) nor Presentation Time (100 vs. 500 vs. 1000 ms) had a significant influence on the timing of gaze shifts toward the target stimulus.

**Table 4:** Fixed effects of the linear mixed-effects model for gaze inflection time in experiment 1b

Fixed Effect	Estimate	CI 95%		df	t	*p*
		Lower	Upper			
Intercept	217.66	162.52	272.81	31.24	7.51	< 0.001
Congruency (Incongruent)	−40.96	−125.36	43.44	37.81	−0.92	0.361
Presentation Time (500 ms)	78.82	−3.11	160.75	33.51	1.87	0.071
Presentation Time (1000 ms)	69.30	−34.07	172.67	71.23	1.27	0.217
Congruent ✻ Presentation Time (500 ms)	12.86	−88.80	114.52	65	0.24	0.811
Congruent ✻ Presentation Time (1000 ms)	40.49	−61.18	142.15	65	0.76	0.453
Random effect	**Random slope**	**Variance**	**SD**	**Correlation**		
Participant	Intercept	1805	42.49			
	Task type (incongruent)	7573	87.02	−0.54		
	Presentation time (500ms)	5073	71.23	0.99	−0.48	
	Presentation time (1000ms)	21,700	147.31	−0.03	0.14	0.08
Residual		9968	99.84			

The intercept represents the reference condition, which includes the neutral-related predictor and the 100 ms presentation time. All other estimates should be interpreted relative to this baseline. Bolded p-values represent significant effects. Abbreviation: *CI* confidence interval, *df* degrees of freedom, *SD* standard deviation

## Discussion

This first experiment investigated whether attentional avoidance occurs in anticipation of threatening content. Overall, participants learned to associate the threatening content with their corresponding predictor cues, which resulted in anticipatory attentional avoidance. Behaviorally, in Experiment 1a, slower reaction times were observed when the target appeared in the same location as the threat (congruent condition) compared to when it appeared in a different location (incongruent condition). However, in Experiment 1b, this effect was observed only with longer presentation times (500ms and 1000ms). These findings are consistent with previous research (Chen et al. 2002; Garner et al. 2006b; Koster et al. 2005; Mogg et al. 2004a, b), as the effect was more consistently demonstrated with longer presentation times. To detect this effect at shorter presentation times, it is possible that we require a higher number of trials per participant. Eye-tracking data further suggested that during the presentation of the predictor cues, participants’ gaze was directed further away from the location of the anticipated threat location, especially during moderately long presentation times. The threat effect was more pronounced in the 500 ms condition compared to the shorter (100 ms) presentation time, which is consistent with previous findings that attentional avoidance tends to emerge after 500ms (Chen et al. 2002; Koster et al. 2006a, b) Together, these findings suggest that participants were attempting to avoid the location of a potential threat, which in turn delayed their ability to focus attention on the target. However, following the presentation of the predictive stimuli, no significant difference was found in the latency of initial gaze shifts toward the target stimulus. Therefore, the difference in reaction time is possibly due to participants’ need to redirect gaze from a location farther away from the target stimulus, rather than a delay in initiating the shift.

The delayed RTs in the congruent, compared to the incongruent condition, may reflect a conflict between bottom-up and top-down processing (Bidet-Caulet et al., 2015; Ligeza et al., 2017). The appearance of the predictor cue would prompt avoidance of the associated spatial location, while the execution of the task would require attending to the same spatial location. Another possible explanation could be the occurrence of inhibition of return (IOR). During inhibition of return (Itti & Koch, 2001; Klein, 2000; Posner et al., 1985), following a rapid, automatic fixation on a threat or potential threat, that specific spatial position is inhibited, meaning that the gaze returns to that position only after a certain delay. Slower reaction times may thus reflect an inhibitory process: participants may have involuntarily tried to avoid the location of the potential threat and had to inhibit this tendency to complete the task.

However, the results of the eye-tracking analysis did not fully support this possibility. The absence of a significant difference in the onset of gaze movement (See Fig. 4. and Table 5.) may suggest that this was not the primary mechanism. Instead, the delay in response may have been due to participants having to return to the target from a greater distance, rather than difficulty from competing information (‘I avoid it because it is threatening’ and ‘I look there because I have to find it’).Additionally, the first fixation occurred more quickly outside the AOIs (See Supplementary Material 1.) than on the predictive stimulus associated with threatening content. According to the definition of IOR (Itti & Koch, 2001; Klein, 2000; Posner et al., 1985), however, we would expect a rapid initial fixation on the threatening stimulus, followed by avoidance behavior.

Taken together, our results suggest that IOR might not apply in our study, rather participants tend to avoid the threat-related spatial positions. To further support this contention, a second experiment was conducted in which the spatial location of the predictor cue and the target stimulus were separated. If avoidance behavior was indeed driving the effect, this should result in faster detection of the target stimulus in our second experiment.

## Experiment 2

Experiment 2 used the same modified version of the cVPT paradigm as was used in Experiment 1. In this version, however, each trial presented a single predictor cue that appeared at the center of the screen, rather than displaying two cues in the corners. In picture trials, the cue was followed by a single picture to establish an association between the cue and its content. In stimulus trials, the target and foil stimuli remained in the corners of the screen, as in Experiment 1. This modification separated the spatial location of the predictor cue and the target stimuli. Experiment 2b served as a replication and an extension of Experiment 2a, incorporating eye-tracking to monitor participants’ eye movements and gaze location over time. We hypothesized that (H1b) participants would locate the target more quickly following the threat-related predictor cue, reflecting an attempt to avoid the location of the predictor associated with the threatening content. Similar to Experiment 1, we hypothesized that (H2) participants’ gaze would be positioned farther away from the location of the potential threat. Lastly, we predicted that (H3b) attentional shifts towards the target would be faster following a threat-related predictor cue compared to a neutral-related predictor cue.

## Method

### Participants

The sample size for Experiment 2a for the within‑subjects Valence (Threat, Neutral) × Presentation Time (100ms, 500ms, 1000ms) interaction in the 2 × 3 rmANOVA was based on the same a priori estimation as in Experiment 1a. Data collection was carried out in one-week increments until the required sample size was reached or exceeded. The final sample consisted of 51 undergraduate students (35 females, mean age = 22.1, SD = 3.20). For Experiment 2b, the sample size required for the analysis was determined based on the results of Experiment 2a (f = 0.45, 1-β = 0.95, *r* =.5), and the analysis indicated a required minimum sample size of 10. The final sample consisted of a total of 22 undergraduate students (15 females, mean age = 20.8 years, SD = 1.56). The analysis of eye-tracking data followed the same statistical approach as in Experiment 1b, including the use of linear mixed-effects models and post hoc power analysis.

All participants reported normal or corrected-to-normal vision. Based on self-report, none of the participants in our study had been diagnosed with or treated for phobia or other psychiatric disorders. All participants were recruited through university mailing lists and were paid 5000 HUF for their participation. Our research was approved by the Hungarian United Ethical Review Committee for Research in Psychology (approval number: EPKEB 2024-084) and was conducted following the guidelines of the World Medical Association Code of Ethics (Declaration of Helsinki). All participants were informed and written informed consent was obtained.

### Stimuli procedure and apparatus

**Fig. 6:**
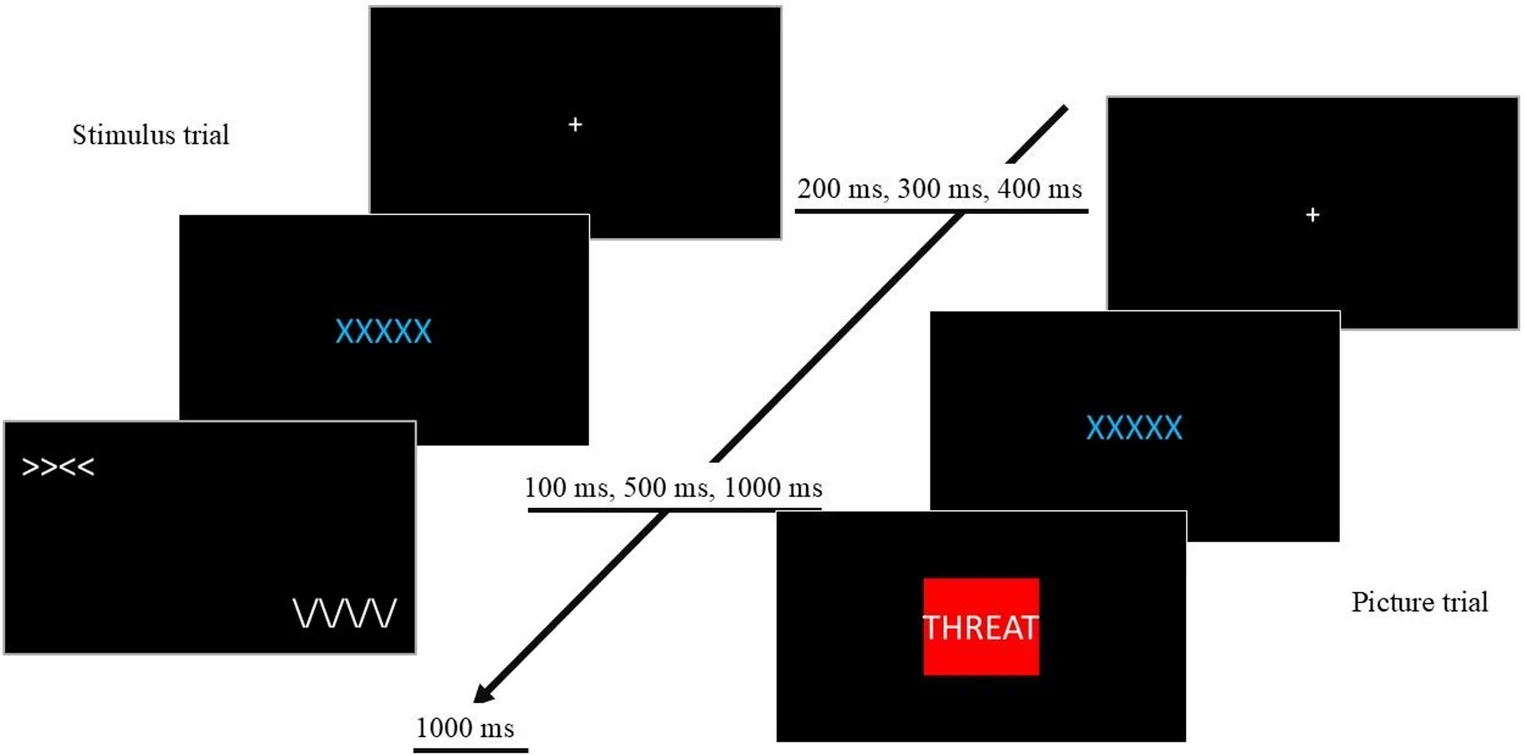
The cVPT paradigm used in Experiment 2. For both trial types, one of the predictive stimuli was displayed at the center of the screen. In picture trials, the image associated with the content of the predictive stimulus (threatening or neutral) was presented centrally following the predictive cue. In stimulus trials, as in the first paradigm, the target (>><<) and foil (\/\/\/or/\/\/\) stimuli were displayed in diagonal pairs on the screen

Although the paradigm for this study was based on the one used in Experiment 1, several modifications were introduced, as shown in Fig. 6. (1) In both picture and stimulus trials, only one predictor cue was presented, (2) which was positioned at the center of the screen. (3) In picture trials, only one picture was presented, also at the center of the screen. In stimulus trials, the target stimulus (>><<) and foil (\/\/\/or/\/\/\) were arranged as in Experiment 1. Experiment 2 consisted of 288 trials in total, evenly split across the two trial types (picture and stimulus). We reduced the number of trials because one of the predictor cues was always presented at the center of the screen. In contrast to Experiment 1, where both cues could appear at four possible locations and required position counterbalancing, the modified design removed the need to control for spatial position. The trials were presented in randomized order in a single testing session, which lasted approximately 15 min. The procedure for both Experiment 2a and 2b mirrored that of Experiment 1a and 1b, respectively, and was carried out using the same apparatus in both cases.

### Statistical analysis

The same statistical analyses conducted for Experiment 1 were applied to Experiment 2. Median RTs were calculated for each participant and for each factor. In Experiment 2b, eye-tracking measures included the distance (in pixels) between participants’ gaze and the location of the threat-related predictor cue, as well as the time elapsed between target presentation and the initiation of gaze movement toward the target location. In both Experiments 2a and 2b, for the behavioral results, outlier trials were identified and removed and defined as those exceeding the ± 2.5 standard deviations from the group median. This resulted in the removal of less than 5% of the data collected. For the behavioral data in both Experiments 2a and 2b, a 2 × 3 rmANOVA was conducted to test the effect of Valence (threat, neutral) and Presentation Time of the predictor cue (100, 500, 1000 ms) as within-subject factors. To break down significant interaction between the two factors, follow-up rmANOVAs per factor were used. Tukey-corrected post hoc tests were conducted to follow up on significant main effects. The assumption of normality was not violated, and absolute values of skewness and kurtosis did not exceed 2 for any factors. Only RTs from correct trials were analyzed, which resulted in the removal of less than 5% of the data collected. Split-half reliability was assessed using Spearman-Brown correction, which indicated good reliability (see Supplementary Material 2) Movement onset time was determined by detecting inflection points in the gaze trajectory. The data were smoothed using a 300‑ms Exponentially Weighted Moving Average (EWMA) to reduce short-term fluctuations while preserving overall trends (Schat et al., 2024; Smit et al., 2023). A subsequent 200‑ms sliding window was applied to identify inflection points, defined as stable changes in the direction of distance increase or decrease. Smoothing was used solely for inflection point detection; all distance comparisons were performed on unsmoothed data.

For the eye-tracking data, a LME model was performed with distance from the threat-related predictor as the dependent variable, the Presentation Time of the predictor (100, 500, 1000ms), and Time (continuous; from 150ms prior to the predictor cue offset) as within-subject fixed effects. An additional LME analysis was conducted with the time of movement onset as the dependent variable and the Valence (threat or neutral) and Predictor presentation time (100, 500, 1000ms) as a within-subject fixed effects. For both models, participants were included as a random effect. Our datasets (including computed study variables) are available on the Open Science Framework: https://osf.io/ej2g7/.

## Results

### Behavioral results

**Fig. 7:**
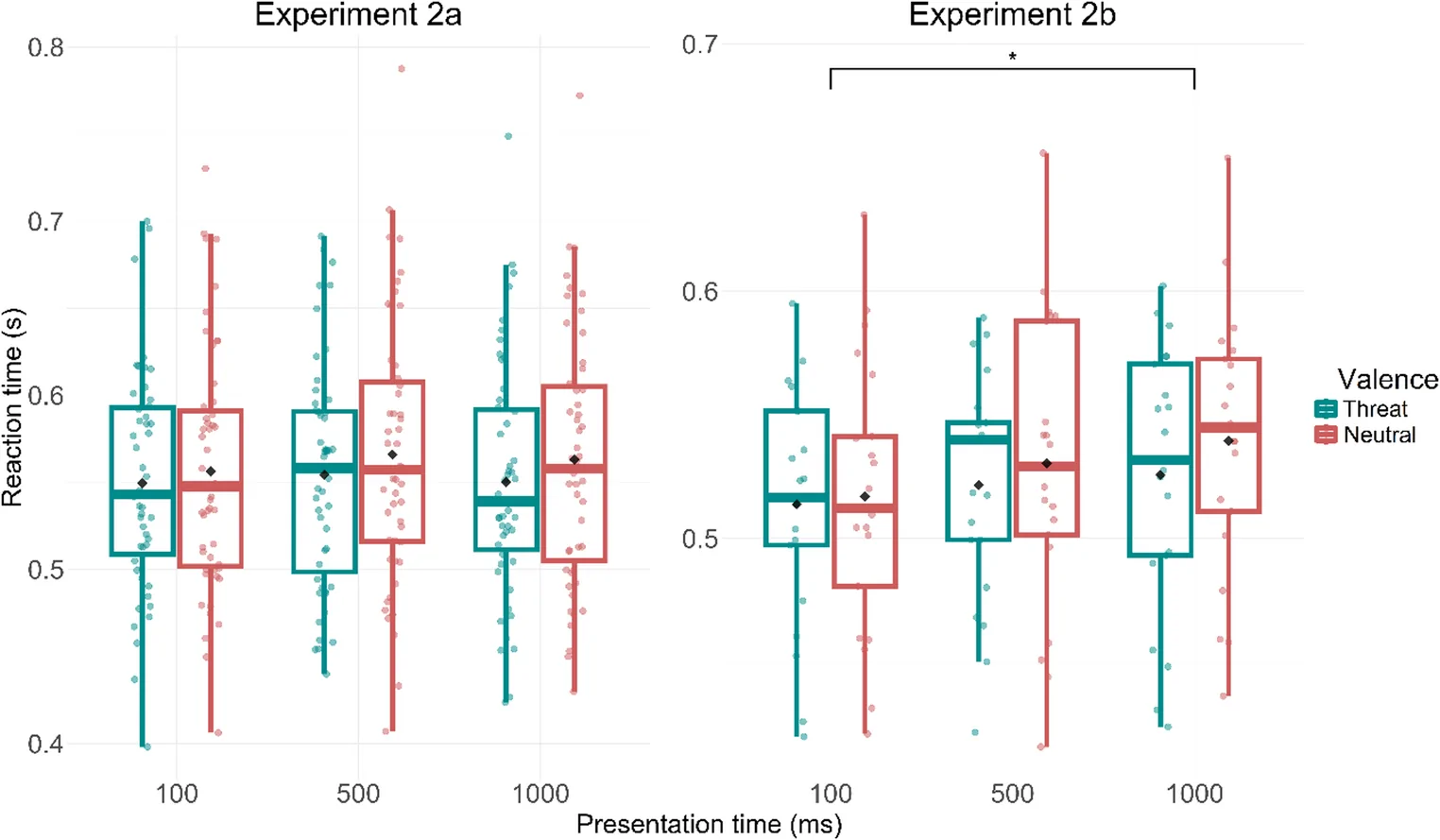
Reaction times (in seconds) for Experiment 2a (left panel) and for Experiment 2b (right panel). Median reaction times (Y-axis) are presented as a function of predictor cue presentation time (X-axis) and Valence. The horizontal middle lines within the boxes indicate the median value, while the black diamonds represent the mean value. The ends of the boxes indicate the upper (Q3) and lower (Q1) quartiles. The lines extending from the boxes show the range of values within Q3 + 1.5 × IQR to Q1–1.5 × IQR, representing the highest and lowest values, excluding outliers. Transparent dots show the median reaction time for each individual. * *p*<.05

RTs in Experiment 2a were analyzed to test the prediction (H1b) that participants would respond faster when attempting to avoid the location of the predictor cue associated with the threatening content. Figure 7 presents the descriptive statistics for these comparisons, and detailed statistical results are presented in Table 5. A significant main effect of Valence was observed, with participants locating the target faster in the congruent condition than in the incongruent condition. Neither the main effect of Presentation time nor the interaction between Valence and Presentation time were significant. These results align with our hypothesis (H1B), as participants showed faster target detection in the congruent condition compared to the incongruent condition.

**Table 5:** Repeated measures ANOVA and post hoc results for Experiment 2a and 2b for reaction times. Post hoc pairwise comparisons are reported only when the main effect or the interaction is significant. Significant main effects were followed up with Tukey-corrected pairwise comparisons, whereas significant interactions were examined using follow‑up ANOVAs

Effect	Comparison	df	F/t	*p*	η²*p*
*Experiment 2a*					
Valence		1, 50	10.08	**0.003**	0.17
Presentation time		2, 100	1.77	0.175	0.03
Valence✻ Presentation time		2, 100	0.51	0.605	0.01
*Experiment 2b*					
Valence		1, 20	4.65	**0.043**	0.19
Presentation time		2, 40	8.42	**< 0.001**	0.30
	100–500	20	−2.28	0.082	
	100–1000	20	−4.02	**0.002**	
	500–1000	20	−1.78	0.202	
Valence✻ Presentation time		2, 40	0.56	0.575	0.03

Bolded p-values represent significant effects

RTs in Experiment 2b were then analyzed. Figure 6 presents the descriptive statistics for these comparisons and detailed statistical results are presented in Table 5. A significant main effect of Valence was also observed indicating that participants located the target faster in the threat compared to the neutral condition. Neither the main effect of Presentation time nor the interaction between Valence and Presentation time reached significance. These findings are also consistent with our original prediction and mirror the results of Experiment 2a, further demonstrating faster target detection in congruent versus incongruent conditions.

### Eye-tracking results

**Fig. 8:**
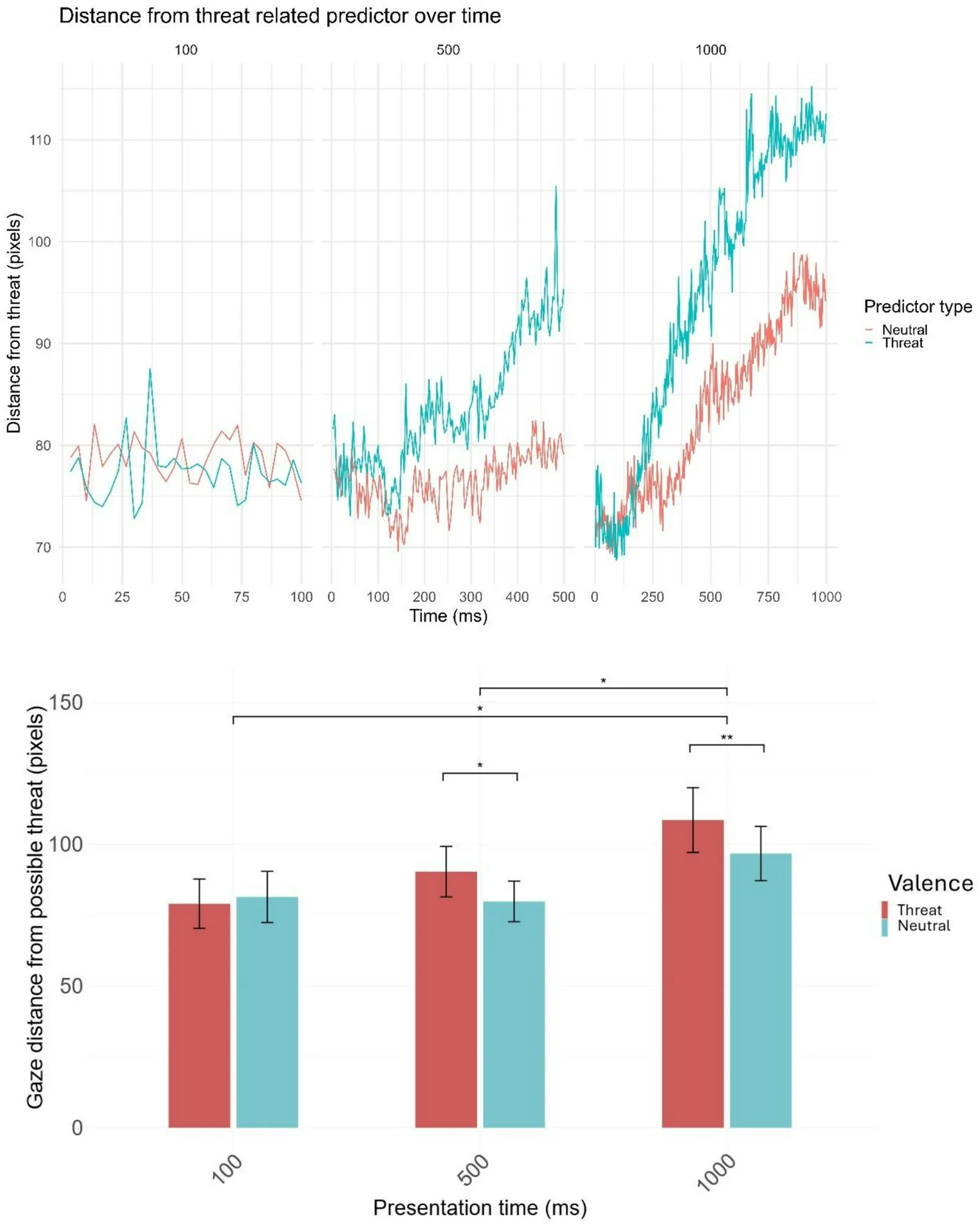
*Top panel*: Gaze distance from the location of the predictor stimulus over time, presented as a function of cue presentation duration (100, 500, 1000 ms). Panels (left to right) correspond to the three presentation times; time is aligned to cue onset and runs until cue offset for each duration. Within each panel, traces show the mean gaze‑to predictor distance (pixels) by Valence (Neutral, Threat); larger values indicate gaze being farther from the threat location; *Bottom panel*: Distance from Threat-Related Predictor Cue as a Function of Presentation Time (in milliseconds) and Predictor Valence (Threat or Neutral).For this panel the time window corresponds to the 150 ms interval preceding cue offset * *p*<.05; **p<.001

Next, the prediction (H2) that participants’ gaze would be directed farther away from the location of the possible threat was examined. The LME’s fixed effects results are summarized in Table 6, and average gaze distances in the different conditions are presented in Fig. 8. No differences between the congruent and incongruent conditions were found. However, a significant interaction was present between Presentation time and Valence, as well as significant differences between Presentation Times.

**Table 6:** Fixed effects of the linear mixed-effects model for gaze distance in experiment 2b

Fixed Effect	Estimate	CI 95%		df	t	*p*
		Lower	Upper			
Intercept	80.76	63.04	98.474	16.08	8.94	< 0.001
Valence (Threat)	−2.42	−10.24	5.400	17	−0.607	0.552
Presentation Time (500 ms)	−1.55	−14.01	10.901	16	−0.244	0.81
Presentation Time (1000 ms)	15.29	1.16	29.429	16	2.121	**0.0498**
Time window	0.008	−0.0003	0.017	4519	1.881	0.0601
Valence ✻ Presentation Time (500 ms)	12.845	11.05	14.638	4519	14.046	**< 0.001**
Valencet ✻ Presentation Time (1000 ms)	14.25	12.46	16.043	4519	15.583	**< 0.001**

The intercept represents the reference condition, which includes the neutral-related predictor and the 100 ms presentation time. All other estimates should be interpreted relative to this reference. Bolded p-values represent significant effects. Abbreviations: *df* degrees of freedom, *SE* standard error

Results indicated no significant difference in gaze distance from the location of the predictor cue at the 100 ms and 500 ms presentation time. However, at 1000 ms, gaze distance from the predictor cue significantly increased compared to the 100 ms presentation time, with an average difference of 15.29 pixels. This suggests that longer presentation times may allow participants to shift their gaze further away from the predictive cue location. Post hoc tests further revealed a significant increase in gaze distance at 1000 ms compared to 500 ms (*t*(16) = − 3.090, *p* =.018).

The interaction terms between Valence and Presentation Time were both significant. Post Hoc tests showed a significant difference in gaze distance between the threat and neutral conditions at 500ms (*t*(17) = − 2.61, *p* =.018), and at 1000 ms (*t*(17) = − 2.97, *p* =.009). This suggests that for both presentation times, greater gaze distance from the predictor cue was observed in the threat condition, compared to the neutral condition. In contrast, no significant difference was found at 100 ms between the conditions (*t*(17) = 0.61, *p* =.552).

Random effects indicated that the standard deviation of the random intercept for participants was 37.19, showing variability in participants’ average performance across trials. The residual standard deviation was 12.69, representing within-participant variability not accounted for by the model. These results suggested that, although participants differed in their average responses, most of the unexplained variability was attributable to trial-level fluctuations within individuals.

Consistent with our prediction (H2), participants’ gaze tended to deviate further from the location of the threat-related predictor cue, indicating an avoidance pattern toward the location of the possible threat.

**Fig. 9:**
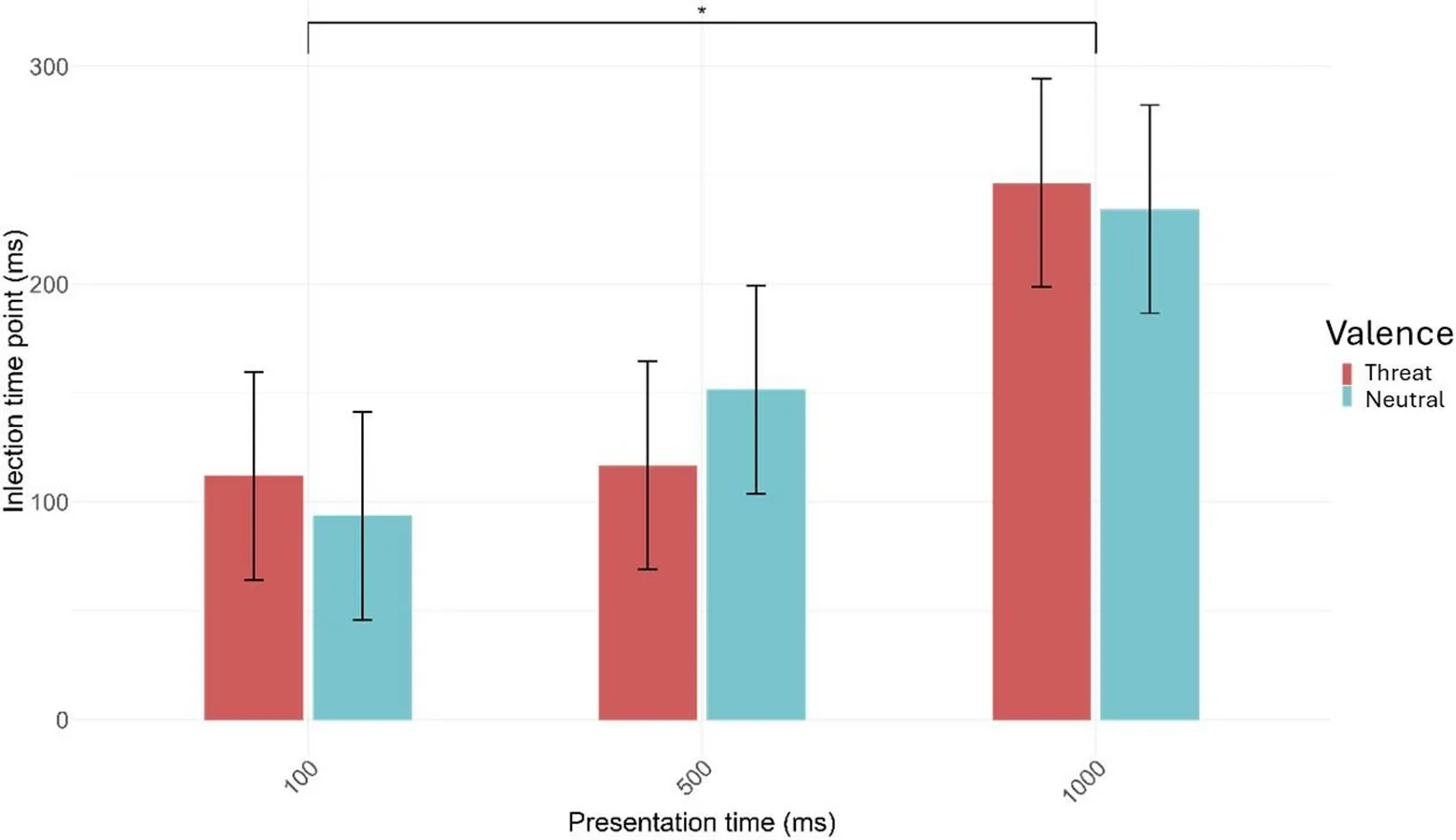
Inflection time point (in milliseconds) of the gaze movement toward the target stimuli as a function of presentation time and predictor valence. * *p*<.05

To test hypothesis 3b (H3b), which predicted that attentional shifts towards the target would be faster following a threat-related predictor cue (compared to a neutral-related predictor cue), an LME model was conducted. The LME’s fixed effects results are summarized in Table 7, and average distances in the different conditions are presented in Fig. 9. No interaction effect or differences between the Threat and Neutral conditions were found.

**Table 7:** Fixed effects of the linear mixed-effects model for gaze inflection time in experiment 2b

Fixed Effect	Estimate	CI 95%		df	t	*p*
		Lower	Upper			
Intercept	93.71	−1.28	189	81.54	1.96	0.054
Valence (Threat)	18.28	−102.73	139	80	0.30	0.765
Presentation Time (500 ms)	57.96	−63.06	179	80	0.95	0.345
Presentation Time (1000 ms)	140.77	19.75	262	80	2.31	**0.024**
Threat ✻ Presentation Time (500 ms)	−53.03	−224.17	118	80	−0.62	0.540
Threat ✻ Presentation Time (1000 ms)	−6.22	−177.37	165	80	−0.07	0.943

The intercept represents the baseline condition, which includes the neutral-related predictor and the 100 ms presentation time. All other estimates should be interpreted relative to this baseline. Bolded p-values represent significant effects. Abbreviations: *df* degrees of freedom, *SE* standard error

The analysis indicated no significant difference in gaze initiation time between the 100 ms and 500 ms Presentation times. However, the 1000 ms Presentation time was associated with a significantly delayed gaze initiation response compared to the 100 ms Presentation time, with an increase of 140.77 ms. Post hoc tests further showed a significant difference between the 500 ms and 1000 ms Presentation times (*t*(80) = 2.46, *p* =.042), indicated that longer predictor cue durations resulted in slower initiation of gaze movement toward the target.

Random effects indicated that the standard deviation of the random intercept for participants was 85.6, reflecting individual differences in average performance across trials. The residual standard deviation was 177.7, representing the within-participant variability that was not explained by the model. These values suggest that, while participants differed in their overall gaze initiation times, the majority of the unexplained variability was due to trial-to-trial fluctuations within individuals.

Overall, these findings suggest that longer predictor cue presentation times, particularly 1000 ms, significantly influenced the timepoint of attentional shift, while the type of Predictor (Threat-related vs. Neutral-related) and its interaction with presentation time did not have any significant effects. These results did not support Hypothesis 3b, which predicted faster initiation of gaze movement toward the target in the Threat condition.

## Discussion

Overall, the results of Experiment 2 were consistent with those of Experiment 1. Behaviorally, participants located the target faster when it was preceded by a threat-related compared to neutral-related cue. One possible explanation for this could be the appearance of an IOR, because if the central area was inhibited, the target could be located more quickly at the peripheral areas of the screen. Another explanation could be that the threatening content predictor increased the level of arousal. This optimal increase in the arousal could lead to faster detection of the target stimuli (Beerendonk et al., 2024; Zsido et al., 2020; Zsidó, 2024; Zsidó et al., 2024). The eye-tracking data showed that the participant’s gaze was directed farther from the threat-related cue compared to the neutral-related cue, indicating a tendency to avoid the center of the screen. This avoidance likely facilitated more efficient exploration of the outer part of the screen, leading to faster target detection. Notably, the threat effect was most prominent at the 1000 ms presentation time, which supports the possibility of attentional avoidance (Chen et al. 2002; Koster et al. 2006a, b). In contrast, no significant differences were found in the initiation time of attentional shifts between threat- and neutral-related cues, suggesting that cue valence did not influence the onset of gaze movement. Furthermore, when comparing the time of first fixation, we found that there were no significant differences on the threat-related and neutral related predictor cue, and there were also no significant differences on the time of first fixation outside of the predictor cue (See Supplementary Material 1.). This could also mean that the predictive stimulus associated with threatening content did not elicit a faster automatic fixation than the one associated with neutral content.

Together, the results of Experiment 2 suggest that participants were able to reliably associate the appropriate predictive stimulus with the possibility of threatening content. Furthermore, both behavioral and eye-tracking results confirmed that participants tended to avoid the spatial location associated with the potential threat, reflecting a pattern of anticipatory attentional avoidance. These findings also support the interpretation that the response delays observed in Experiment 1 were driven by avoidance, rather than by a conflict between bottom-up and top-down processes.

### General discussion

The aim of this study was to investigate whether avoidance, as a primary attentional bias, tends to occur during the anticipation of threat. In Experiment 1, the predictor cues (one associated with threatening content, the other with neutral content) appeared in the same location as the target and the foil. In Experiment 2, the predictor cues appeared in the center of the screen, while the target and foil were in the corners of the screen. We hypothesized that a cue associated with threatening content would lead to the avoidance of its location. Our behavioral and eye-movement findings both appear to support this claim. Across both experiments, participants successfully associated the threatening content with the predictor cue. The results showed that participants tended to avoid the spatial position of the threat-related predictor cue, leading to slower target detection in Experiment 1, and faster detection in Experiment 2. However, there was no effect on the initiation of the gaze toward the target stimuli which likely also supports the possibility that avoidance appears as a primary attentional process.

Participants tended to avoid the location of the predictor cue associated with the threatening content. In both paradigms, a clear difference emerged between the two conditions: following the presentation of the threat-related predictor cue (compared to the neutral-related one), participants were slower to detect the target stimulus in Experiment 1 and faster in Experiment 2. The behavioural results could be partially explained by an inhibition of return. Participants may automatically fixate on the threat-related predictor, and then avoid that spatial position for some time (Itti & Koch, 2001; Klein, 2000; Posner et al., 1985). In Experiment 1, this may have delayed reaction times in the congruent condition, whereas in Experiment 2, the faster responses may also reflect increased arousal induced by the appearance of a possible threat. Multiple studies (Beerendonk et al., 2024; Zsido et al., 2020; Zsidó, 2024; Zsidó et al., 2024) have suggested that an optimal increase in arousal can improve visual search performance, leading to faster detection of target stimuli. However, the behavioral and eye-tracking results from Experiments 1 and 2 together suggest that participants anticipated the appearance of the threat and tried to avoid the spatial position where threatening content was expected to occur. Supporting this interpretation, we found that during the presentation of the predictive stimuli, participants’ gaze was directed farther from the threat-associated predictor than from the neutral-associated one. Previous research has shown that the processing of threatening content often overrides the processing of other stimuli (Koster et al. 2006a, b; Mogg et al., 2004b; Pflugshaupt et al., 2005). Notably here, the anticipatory stimulus did not present any threatening content but merely predicted the possible spatial position of the upcoming threat. The effect of the threat is therefore not limited to its actual presence but can also arise when threat is anticipated (Bastiaansen & Brunia, 2001; Brinkmann et al., 2017; Gladwin, 2019).

In Experiment 1, if participant’s gaze location was farther away from the threat-predicting stimulus, they would have to return to that spatial position in congruent trials to respond appropriately, which may be a contributing factor to their slower RTs. By contrast, in Experiment 2, avoiding the center of the screen (where the predictor appears) allowed participants to more easily detect target stimuli appearing on the outer areas of the screen. This avoidance behavior may occur because the location of the threatening stimulus was already known (through learning what was signaled by the predictor cue), and therefore the exploration of the surroundings provided more relevant information to make the best possible decision when the threatening stimulus was presented (Koster et al., 2006b; Pflugshaupt et al., 2005).

We found no difference in the time at which participants started to shift their gaze toward the target stimulus after a threat-related cue compared to a neutral-related cue. Thus, the effect of the content associated with the anticipatory stimulus was no longer detectable during the attentional shift. This may be due to participants’ peripheral perception (Carretié et al., 2017; Gao et al., 2017), as they were able to detect that the threatening content was not present, signaling that they could proceed with the task. Since the threatening image was not present, participants may have been able to release the inhibition on that spatial position (a bottom-up process) and were able to focus on the execution of the task (a top-down process). This could also suggest that if the predictive stimuli influenced the attentional processes during the task, this effect may have occurred at the covert level (Calvo et al., 2015a, b; Cave & Batty, 2006; Fernández-Martín et al., 2017).

In both the first and second experiments, our behavioral results showed greater differences in reaction times for the 500 ms and 1000 ms presentations than for the 100 ms presentation time condition. The same pattern was observed in the eye-tracking data, as participants’ gaze was directed farther away from the stimulus predicting threatening content during the 500 ms and 1000 ms presentations in both trial types. Previous research has shown conflicting results regarding the timing of attentional avoidance. Some studies have suggested that avoidance can occur even at short presentation times (Sagliano et al., 2014; Waters et al., 2007), while others argued that it emerges only at longer presentation times (Chen et al. 2002; Garner et al. 2006b; Koster et al. 2005; Mogg et al. 2004a, b). Our findings support the notion that attentional avoidance tends to appear at longer (500ms or longer) presentation times. This may be because participants require sufficient time to process the content of the anticipatory stimulus and initiate avoidance behavior. It was previously suggested (Cisler et al., 2009) that avoidance may appear after 500 or 1000ms as only a secondary process, and after attentional attraction or capture. However, based on our eye-tracking results, we found no evidence of earlier competing processes. Instead, our results suggest that avoidance may reflect a distinct attentional bias.

#### Limitations

The effective sample sizes in the eye tracking experiments were modest. Even though trial counts were high, increasing participant numbers in future studies could strengthen precision and the robustness of our results. Additionally, the present experimental arrangement did not allow us to disentangle stimulus valence from arousal. Future studies should therefore use designs in which images varying in both valence and arousal are presented in separate blocks, allowing a clearer dissociation of these factors. Finally, our stimulus set focused on a single threat domain, medical related stimuli (e.g., syringes, surgery, blood draw), which may limit generalizability to other threat types (e.g., animals, guns). Subsequent studies should broaden stimulus categories and directly compare domains within participants to evaluate whether anticipatory avoidance generalizes across threat classes. Furthermore, possible explanations for our findings could be related to covert attention. It is possible that, in Experiment 1, participants were able to process the content of the predictor cues due to peripheral vision. The methods used in the current study did not allow us to investigate these processes. Additional studies using more detailed measures (e.g. EEG) are needed to explore the role of covert attention.

## Conclusion

Despite these limitations, our findings suggest that avoidance, likely as a primary attentional bias, may be elicited by threat anticipation. In two experiments, participants consistently avoided the spatial location of a predictive cue associated with threatening content, as reflected in both reaction times and eye movement measures. Importantly, while participants’ gaze moved away from the threat-predicting cue, there was no effect on the initiation of gaze to the target, reinforcing the idea that avoidance occurs at an early stage of attentional processing. Crucially, we observed this bias in a non-clinical population, demonstrating that threat anticipation alone can shape attentional allocation. Regarding viewing distance, the random effects results showed heterogeneity in gaze movement at longer presentation time (1000ms). This variability suggests that individual viewing strategies can differ in longer presentation time; this heterogeneity should be considered in future experimental designs. These findings have implications for understanding how avoidance mechanisms function in typical cognition and how they may be exaggerated in anxiety disorders. Future research should explore the neural mechanisms underlying anticipatory avoidance and its potential role in clinical populations.

## Supplementary Information

Below is the link to the electronic supplementary material.


Supplementary Material 1 (DOCX 108 KB)



Supplementary Material 2 (DOCX 167 KB)



Supplementary Material 3 (DOCX 21.3 KB)


## Data Availability

Our datasets (including computed study variables) are available on the Open Science Framework: https://osf.io/ej2g7/.

## Footnotes

1 The Geneva Affective Picture Database (GAPED; Dan-Glauser & Scherer, 2011), The Nencki Affective Picture System (NAPS; Marchewka et al., 2013), EmoMadrid (Carretié et al., 2019).

2 During the review process, it occurred to us that this phenomenon might be explained by the process of inhibition of return (IOR). To test this theory, we conducted further analyses of fixation probability and time to first fixation. Comparisons along the relevant AOIs suggest that IOR might not apply to our study; for details, see Supplementary Material 1.
