# Supplementary material for: Anticipatory attentional avoidance in learned threat associations
Source: Psychol Res. 2026 Jul 15;90(4):136. doi: 10.1007/s00426-026-02342-1 (PMC13372855; doi:10.1007/s00426-026-02342-1)
Supplement: Supplementary file 1 — Supplementary Material 1 (DOCX 108 KB) [file 426_2026_2342_MOESM1_ESM.docx]

**Supplementary Material S1. Fixation‑Probability (AOI) Analyses — Experiments 1b and 2b**

This Supplementary Material file reports AOI‑based fixation probabilities during the cue presentation window to complement the distance metrics presented in the main text.

Areas of interest (AOIs) were predefined rectangular regions on the display used to quantify gaze allocation (e.g., fixations) to specific on‑screen locations. In Experiment 1b, two predictor stimuli were presented on diagonal positions; therefore, separate AOIs captured fixations to the Threat‑predictor and Neutral‑predictor locations, in addition to a central Fixation‑cross AOI. In Experiment 2b, the predictor stimulus appeared centrally; therefore, we defined a Central‑predictor AOI and a central Fixation‑cross AOI. The Outside AOI represents all remaining screen areas not covered by any of the defined AOIs (i.e., the complement of the AOIs). All AOIs were identical in size across conditions and were centered on the corresponding stimulus locations.

**Statistical analysis.**

Experiment 1b: 4x3 repeated‑measures ANOVA with AOI (Fixation cross, Threat, Neutral, Outside) × Presentation time (100, 500, 1000 ms).

Experiment 2b: 3x2x3 repeated‑measures ANOVA with AOI (Fixation cross, Predictor position, Outside), Valence (Threat, Neutral), Presentation time (100, 500, 1000 ms).

Effect sizes are partial eta squared (η_p_²). Tukey‑adjusted post hoc comparisons unpack significant main effects and interactions. Follow‑up ANOVAs (simple effects) are reported by AOI within each presentation time and by presentation time within each AOI.

Table S1.1: Repeated measures ANOVA results for Experiments 1b.

| Effect | Comparisons | df | F/t | p | η²p |
| --- | --- | --- | --- | --- | --- |
| Presentation time |  | 2,18 | 1.24e-15 | 1.000 | 0.000 |
| AOI |  | 3 | 7.97 | <.001 | 0.470 |
|  | Fix cross – Threat | 9 | 2.91 | 0.07 |  |
|  | Fix cross – Neutral | 9 | 2.81 | 0.08 |  |
|  | Fix cross – Outside | 9 | -0.07 | 1.00 |  |
|  | Threat - Neutral | 9 | 0.37 | 0.98 |  |
|  | Threat - Outside | 9 | -4.2 | 0.01 |  |
|  | Neutral - Outside | 9 | -3.96 | 0.01 |  |
| Presentation time ✻ AOI |  | 6 | 6.60 | <.001 | 0.423 |

*Note: The follow analysis for the interactions is presented Table S1.2.*

Figure S1: Differences in Fixation probability in the different AOIs (Fixation cross, Predictor position, Outside) and Presentation times (100, 500, 1000 ms).


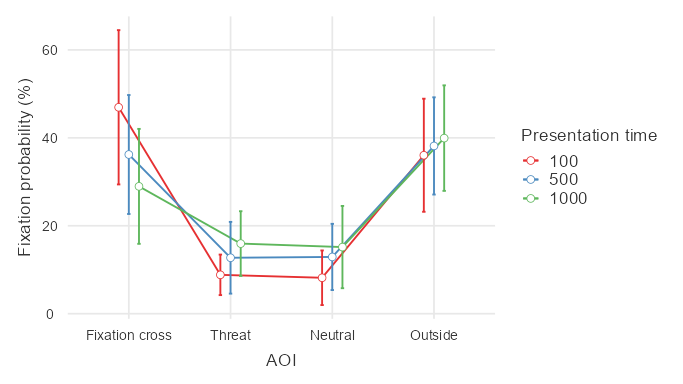


Table S1.2: Follow‑up rmANOVA for the significant interaction (Presentation time ✻ AOI).

| Effect | Comparisons | df | F/t | p | η²p |
| --- | --- | --- | --- | --- | --- |
| 100 ms | AOI | 3, 27 | 11.0 | **<.001** | 0.550 |
|  | Fix cross - Threat | 9.00 | 4.111 | **0.012** |  |
|  | Fix cross - Neutral | 9.00 | 3.986 | **0.014** |  |
|  | Fix cross - Outside | 9.00 | 0.851 | 0.829 |  |
|  | Threat - Neutral | 9.00 | 0.407 | 0.976 |  |
|  | Threat- Outside | 9.00 | -4.796 | **0.004** |  |
|  | Neutral - Outside | 9.00 | -4.580 | **0.006** |  |
| 500 ms | AOI | 3, 27 | 7.11 | **0.001** | 0.441 |
|  | Fix cross - Threat | 9.00 | 2.674 | 0.098 |  |
|  | Fix cross - Neutral | 9.00 | 2.677 | 0.097 |  |
|  | Fix cross - Outside | 9.00 | -0.227 | 0.996 |  |
|  | Threat - Neutral | 9.00 | -0.112 | 0.999 |  |
|  | Threat- Outside | 9.00 | -3.403 | **0.033** |  |
|  | Neutral - Outside | 9.00 | -3.588 | **0.025** |  |
| 1000 ms | AOI | 3, 27 | 4.68 | **0.009** | 0.342 |
|  | Fix cross - Threat | 9.00 | 1.572 | 0.439 |  |
|  | Fix cross - Neutral | 9.00 | 1.541 | 0.454 |  |
|  | Fix cross - Outside | 9.00 | -1.276 | 0.599 |  |
|  | Threat - Neutral | 9.00 | 0.317 | 0.988 |  |
|  | Threat- Outside | 9.00 | -3.185 | **0.045** |  |
|  | Neutral - Outside | 9.00 | -2.939 | 0.066 |  |

*Note:* Bolded p-values represent significant effects

Table S2.1: Repeated measures ANOVA results for Experiments 2b.

| Effects | Comparisons | df | F | p | η²p |
| --- | --- | --- | --- | --- | --- |
| AOI |  | 2,24 | 34.349 | **<.001** | 0.741 |
|  | Fix cross - Middle position (predictor) | 12.0 | -5.07 | <.001 |  |
|  | Fix cross - Outside | 12.0 | 2.65 | 0.052 |  |
|  | Middle position (predictor) - Outside | 12.0 | 9.46 | <.001 |  |
| Valence |  | 1,12 | 1.738 | 0.212 | 0.127 |
| Presentation time |  | 2,24 | 7.911 | **0.002** | 0.397 |
|  | 100 - 500 | 12.0 | -2.70 | 0.047 |  |
|  | 100 - 1000 | 12.0 | -2.82 | 0.038 |  |
|  | 500 - 1000 | 12.0 | -2.84 | 0.037 |  |
| AOI ✻ Valence |  | 2,24 | 0.603 | 0.555 | 0.048 |
| AOI ✻ Presentation time |  | 4,48 | 9.862 | **<.001** | 0.451 |
| Valence ✻ Presentation time |  | 2,24 | 1.408 | 0.264 | 0.105 |
| AOI ✻ Valence ✻ Presentation time |  | 4,48 | 1.244 | 0.305 | 0.094 |

*Note:* Bolded p-values represent significant effects

Figure S2.: Differences in Fixation probability in the different AOIs (Fixation cross, Predictor position, Outside) an Presentation times (100, 500, 1000 ms) and Valence (Threat, Neutral)


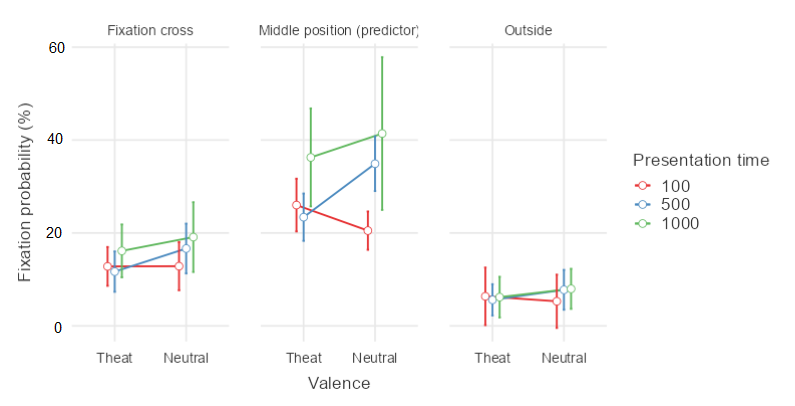


Table S2.2: Follow‑up rmANOVA for the significant interaction (Presentation time ✻ AOI).

| Effect | Comparisons | df | F/t | p | η²p |
| --- | --- | --- | --- | --- | --- |
| AOI |  | 2,24 | 34.35 | **<.001** | 0.741 |
|  | Fix cross - Middle | 12.0 | -5.07 | **<.001** |  |
|  | Fix cross - Outside | 12.0 | 2.65 | 0.052 |  |
|  | Middle - Outside | 12.0 | 9.46 | **<.001** |  |
| Presentation time | | 2,24 | 7.91 | **0.002** | 0.397 |
|  | 100 - 500 | 12.0 | -2.70 | **0.047** |  |
|  | 100 - 1000 | 12.0 | -2.82 | **0.038** |  |
|  | 500 - 1000 | 12.0 | -2.84 | **0.037** |  |
| AOI ✻ Presentation time | | 4,48 | 9.86 | **<.001** | 0.451 |
|  | Presentatiom time - 100ms | 2,24 | 26.7 | **<.001** | 0.690 |
|  | Fix cross - Middle | 12.0 | -5.44 | **<.001** |  |
|  | Fix cross - Outside | 12.0 | 2.37 | 0.084 |  |
|  | Middle - Outside | 12.0 | 7.87 | **<.001** |  |
|  | Presentatiom time - 500ms | 2,24 | 32.3 | **<.001** | 0.729 |
|  | Fix cross - Middle | 12.0 | -5.14 | **<.001** |  |
|  | Fix cross - Outside | 12.0 | 2.41 | 0.078 |  |
|  | Middle - Outside | 12.0 | 9.00 | **<.001** |  |
|  | Presentatiom time - 1000ms | 2,24 | 29.7 | **<.001** | 0.713 |
|  | Fix cross - Middle | 12.0 | -4.43 | **0.002** |  |
|  | Fix cross - Outside | 12.0 | 2.79 | **0.040** |  |
|  | Middle - Outside | 12.0 | 8.04 | **<.001** |  |

*Note:* Bolded p-values represent significant effect
