# Supplementary material for: Anticipatory attentional avoidance in learned threat associations
Source: Psychol Res. 2026 Jul 15;90(4):136. doi: 10.1007/s00426-026-02342-1 (PMC13372855; doi:10.1007/s00426-026-02342-1)
Supplement: Supplementary file 3 — Supplementary Material 3 (DOCX 21.3 KB) [file 426_2026_2342_MOESM3_ESM.docx]

**Supplementary Material S2. Spearman-Brown split-half reliability analyses — Experiments 1b and 2b**

This Supplementary Material presents Split-half reliability results for each behavioral experiment in each condition. The tables report the mean and standard deviation (SD) of reaction times for the two split halves (Half A and Half B), as well as the Spearman–Brown corrected reliability coefficients.

Table 1.: Experiment 1a

| Measure | Half A Mean | Half A SD | Half B Mean | Half B SD | Spearman-Brown coefficient |
| --- | --- | --- | --- | --- | --- |
| Cong 100 ms | 0.619 | 0.069 | 0.612 | 0.067 | 0.966 |
| Cong 500 ms | 0.650 | 0.098 | 0.637 | 0.070 | 0.911 |
| Cong 1000 ms | 0.616 | 0.075 | 0.618 | 0.074 | 0.926 |
| Incong 100 ms | 0.605 | 0.071 | 0.614 | 0.072 | 0.925 |
| Incong 500 ms | 0.634 | 0.080 | 0.635 | 0.087 | 0.923 |
| Incong 1000 ms | 0.618 | 0.069 | 0.612 | 0.079 | 0.936 |
| Cong overall | 0.629 | 0.076 | 0.623 | 0.067 | 0.976 |
| Incong overall | 0.619 | 0.066 | 0.620 | 0.074 | 0.975 |

Table 2.: Experiment 1b

| Measure | Half A Mean | Half A SD | Half B Mean | | Half B SD | | Spearman-Brown coefficient |
| --- | --- | --- | --- | --- | --- | --- | --- |
| Cong 100 ms | 0.553 | 0.081 | | 0.561 | | 0.089 | 0.694 |
| Cong 500 ms | 0.557 | 0.087 | | 0.557 | | 0.077 | 0.939 |
| Cong 1000 ms | 0.547 | 0.064 | | 0.564 | | 0.087 | 0.833 |
| Incong 100 ms | 0.558 | 0.079 | | 0.547 | | 0.069 | 0.929 |
| Incong 500 ms | 0.562 | 0.068 | | 0.558 | | 0.075 | 0.919 |
| Incong 1000 ms | 0.550 | 0.074 | | 0.559 | | 0.075 | 0.778 |
| Cong overall | 0.552 | 0.071 | | 0.561 | | 0.075 | 0.925 |
| Incong overall | 0.556 | 0.068 | | 0.555 | | 0.066 | 0.953 |

Table 3.: Experiment 2a

| Measure | Half A Mean | Half A SD | Half B Mean | Half B SD | Spearman-Brown coefficient |
| --- | --- | --- | --- | --- | --- |
| Cong 100 ms | 0.653 | 0.098 | 0.656 | 0.094 | 0.921 |
| Cong 500 ms | 0.684 | 0.094 | 0.685 | 0.099 | 0.802 |
| Cong 1000 ms | 0.671 | 0.098 | 0.673 | 0.086 | 0.865 |
| Incong 100 ms | 0.654 | 0.106 | 0.658 | 0.100 | 0.968 |
| Incong 500 ms | 0.668 | 0.100 | 0.671 | 0.102 | 0.967 |
| Incong 1000 ms | 0.659 | 0.088 | 0.675 | 0.117 | 0.960 |
| Cong overall | 0.669 | 0.089 | 0.671 | 0.085 | 0.964 |
| Incong overall | 0.661 | 0.095 | 0.668 | 0.103 | 0.990 |

Table 4: Experiment 2b

| Measure | Half A Mean | Half A SD | Half B Mean | Half B SD | Spearman-Brown coefficient |
| --- | --- | --- | --- | --- | --- |
| Threat 100 ms | 0.576 | 0.146 | 0.570 | 0.150 | 0.959 |
| Threat 500 ms | 0.584 | 0.148 | 0.598 | 0.161 | 0.967 |
| Threat 1000 ms | 0.605 | 0.136 | 0.586 | 0.112 | 0.981 |
| Neutral 100 ms | 0.602 | 0.228 | 0.606 | 0.191 | 0.986 |
| Neutral 500 ms | 0.592 | 0.164 | 0.623 | 0.292 | 0.981 |
| Neutral 1000 ms | 0.611 | 0.172 | 0.610 | 0.190 | 0.990 |
| Threat overall | 0.588 | 0.132 | 0.586 | 0.131 | 0.991 |
| Neutral overall | 0.610 | 0.184 | 0.617 | 0.220 | 0.990 |
